# Hairpin inserts in viral genomes are stable when they conform to the thermodynamic properties of viral RNA substructures

**DOI:** 10.1128/jvi.01919-24

**Published:** 2025-03-21

**Authors:** Chanyong Jang, Jason M. Needham, Philip Z. Johnson, Feng Gao, Anne E. Simon

**Affiliations:** 1 Department of Cell Biology and Molecular Genetics, University of Maryland College Park 171289 , College Park, Maryland, USA; 2 Silvec, Inc, Gaithersburg, Maryland, USA; Iowa State University, Ames, Iowa, USA

**Keywords:** RNA structure, positional entropy, RNA plasticity, virus-induced gene silencing, VIGS

## Abstract

Virus-induced gene silencing (VIGS) allows for the rapid targeting of gene expression and has been instrumental in characterizing plant genes. However, foreign sequences inserted into VIGS vectors are rarely maintained for unknown reasons. Citrus yellow vein-associated umbravirus-like virus (CY1) with its solved secondary structure was converted into a VIGS vector to determine why simple hairpins inserted into non-functional, single-stranded locations are not maintained. When CY1 contained foreign hairpins with thermodynamic properties (positional entropy and/or ΔG) differing from those of natural CY1 hairpins, deletions arose within a few weeks of infecting *Nicotiana benthamiana*. In contrast, duplication and insertion of four natural CY1 hairpins (up to 200 nt) into the same locations were retained until plant senescence. Hairpins containing similar conformations and thermodynamic properties as natural hairpins were also retained, as were hairpins that shared thermodynamic properties but were conformationally distinct. By predicting and modulating these thermodynamic properties, a hairpin was retained by CY1 for at least 30 months in citrus. These findings strongly suggest that RNA viruses have evolved to contain substructures with specific thermodynamic properties, and hairpins containing these properties are stable when inserted into non-functional regions of the genome, opening up VIGS for long-lived trees and vines.

**IMPORTANCE:**

Plus-strand RNA plant viruses are used as tools to introduce small interfering RNAs (siRNAs) into laboratory plants to target and silence genes. However, virus-induced gene silencing (VIGS) vectors engineered to contain foreign hairpins or other sequences for siRNA generation are not stable, and the foreign sequences are rapidly lost. We found that foreign sequences are not maintained in an umbravirus-like VIGS vector (CY1) because their physical properties conflict with the innate properties of the CY1 genome’s substructures (i.e., hairpins). When natural CY1 hairpins were duplicated and inserted into locations where previous inserts were rapidly lost, the hairpins were now stable as were unrelated hairpins with the same physical properties. By mimicking the physical properties of the viral genome, one insert was stable for over 30 months. These results suggest that RNA viral genomes have evolved to have specific physical properties, and these properties appear to be similar for other plus-strand RNA viruses.

## INTRODUCTION

Virus-induced gene silencing (VIGS) is a common technique that uses post-transcriptional gene silencing, a process intrinsic to plant antiviral defenses, to suppress the expression of target genes (1). VIGS most often involves engineering a plus-sense +RNA virus to include an RNA sequence complementary to a target mRNA, which is inserted into a location within the viral genome that is not engaged in critical functions such as replication, translation, and movement (2). When VIGS vectors infect a plant cell, highly structured regions of the viral genome are cleaved by dicer-like endonucleases (DCLs) to generate virus-specific 21–24 nt viral siRNAs (vsiRNAs). vsiRNAs are incorporated into RNA-induced silencing complexes where the retained strand along with Argonaute proteins hybridizes to a specific target RNA and induces either sequence-specific degradation or translational repression (3, 4). vsiRNAs are also generated from the entire viral genome by transitivity, whereby host RNA-dependent RNA polymerase 6 (RdRp6) synthesizes fully double-stranded viral RNAs that are processed by DCLs into additional vsiRNAs for transport throughout the plant (1, 5, 6).

Since the first report that a tobacco mosaic virus VIGS vector harboring a sequence complementary to *Phytoene desaturase* (PDS) mRNA silenced the endogenous gene in *Nicotiana benthamiana* (7), over 50 +RNA, DNA, and satellite +RNA viruses have been adapted to serve as VIGS vectors for silencing genes in a wide variety of plants (5). Since VIGS vectors can induce rapid phenotypic responses, they are important laboratory tools for functional genomics and for elucidating defense mechanisms against biotic and abiotic stress (5, 8). VIGS vectors may also serve as important tools for agricultural development (e.g., controlling pathogens and altering traits in field crops and long-lived trees and vines) if symptoms are mild and transmission is negligible. However, the agricultural application of VIGS has been limited due to the rapid deletion of foreign RNA inserts (9, 10). Loss of inserts is often attributed to reduced viral fitness owing to toxic sequences, length of the insert, or inappropriate insertion sites (9–13), with insert removal proposed to be a consequence of viral RdRp recombination. Efforts to stabilize a brome mosaic virus VIGS vector by using computer-predicted single-stranded insertion sites provided partial stabilization of inserts at 7 days post-inoculation (12), and reducing insert size in a cucumber green mottle mosaic virus vector also enhanced insert retention (13). However, these studies only achieved partial insert retention, which is insufficient for use in long-lived agricultural plants.

Viral RNA genomes, as with all RNA molecules, fold to contain multiple substructures (14). Ancel and Fontana (15) theorized that RNA substructures with high plasticity (i.e., substructures that are likely to assume a variety of shapes as their residues have multiple possible pairing partners) evolve to reduce their plasticity, which provides a fitness advantage by reducing the number of conformations that RNA substructures can assume under thermal fluctuations, allowing the most advantageous structure to predominate. Experimental evidence for their hypothesis was provided by Maeda et al. (16), who used an *in vitro* translation-coupled RNA replication system and replicon template derived from Qβ bacteriophage to show that the persistence of RdRp-generated mutations in the replicon population over time significantly improved replication of the template (i.e., increased template fitness), which correlated with decreased RNA plasticity in computationally predicted substructures. In addition, increased activity of the *Tetrahymena* ribozyme following *in vitro* evolution was also accompanied by reduced plasticity (17). These reports suggest an alternative explanation for why foreign sequence inserts are rapidly lost from VIGS vectors: highly evolved, low plasticity viral genomes that are engineered to contain foreign sequence inserts with unsuitable thermodynamic properties rapidly evolve during replication by non-processive RdRp to eliminate the foreign sequences, thus enhancing fitness. If this hypothesis is correct, then understanding the thermodynamic properties of endogenous RNA substructures and applying these properties to foreign RNA sequences inserted into VIGS vectors should lead to greater retention of the inserts.

**Fig 1:**
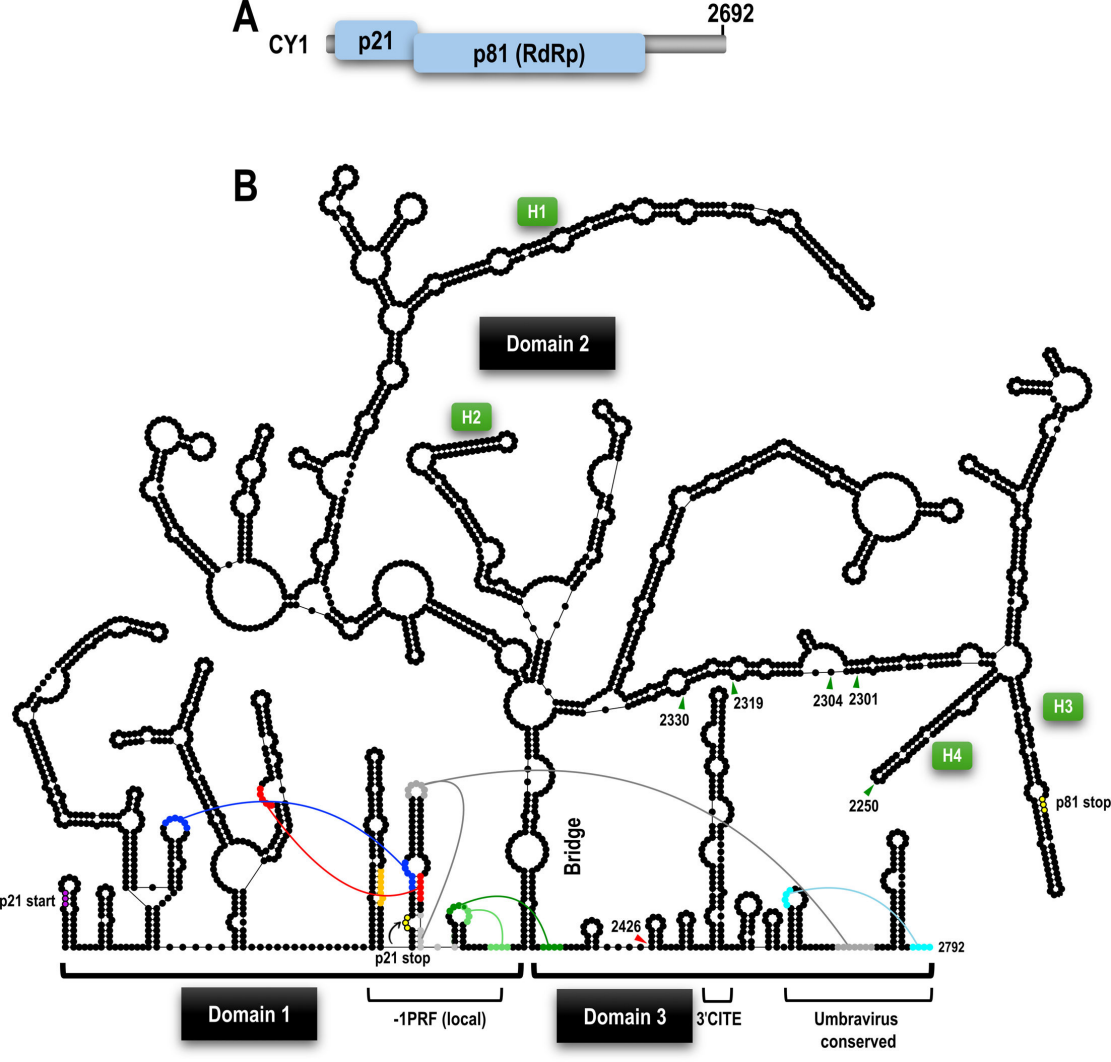
Genome organization and full-length secondary structure of CY1 genomic RNA. (**A**) Genome organization of CY1. p21 in related viruses is a replication-associated protein. The p81 RdRp is synthesized following a −1PRF event. (**B**) Secondary structure of CY1. Domains 1, 2, and 3 are designated, as is the “bridge” that separates domain 2 from the other domains. p21 start and stop codons are indicated, as is the p81 stop codon. Tertiary interactions associated with the −1PRF recoding site or with the 3ʹ terminal hairpins are color coded. Some of these interactions are incompatible with each other and found in alternative conformations. The −1PRF slippery site is in orange. Elements associated with translation (3ʹCITE) and replication (labeled as “umbravirus conserved”) are also indicated. Sites within the 3ʹ UTR in domain 2 that can accommodate inserts are denoted by green triangles. The red triangle in domain 3 denotes a site that could not accommodate inserts due to negative effects on translation. Four hairpins (labeled H1–H4) described in the text are also indicated.

To examine this hypothesis, we chose to study the umbravirus-like virus (ULV) citrus yellow vein-associated virus (CY1; also known as CYVaV). CY1 (2,692 nt) is a +RNA virus with two open reading frames (ORFs) encoding a 21 kDa replication-associated protein and the 81 kDa RdRp, whose synthesis requires a −1 ribosomal frameshift event near the end of the p21 ORF (Fig. 1A). CY1 is unusual in that it does not encode movement or capsid proteins, which are normally required for cell-to-cell and long-distance movement of viral genomic RNAs. Rather, CY1 systemically infects the vascular tissue of a broad range of citrus and non-citrus hosts in the absence of any helper virus by using a host RNA movement protein (18). CY1 also contains an extensive 3ʹ UTR due to a natural loss of a capsid-like protein ORF (encoded in related virus CY2) due to alterations in a subgenomic promoter along with site-specific mutations and two large deletions (18). Importantly, for this study, CY1 is one of the few +RNA viruses with a fully mapped genomic RNA secondary structure (19–23). Additionally, locations of many important functional elements involved in CY1 replication and translation have already been defined (24, 25).

In this report, we describe the conversion of CY1 into a VIGS vector by identifying five locations that can accommodate inserts without losing infectivity. Nearly all hairpins with predicted thermodynamic properties (positional entropy [PE] and/or ΔG) differing from those of natural CY1 hairpins experienced deletions within a few weeks of infiltration. In contrast, duplication and insertion of four natural CY1 hairpins (up to 197 nt) into the same locations were maintained until plant senescence. Hairpins designed to mimic natural hairpins by containing similar conformations and thermodynamic properties were also retained in the CY1 VIGS vector, as were hairpins that shared thermodynamic properties but were conformationally distinct. By predicting and modulating these thermodynamic properties, hairpins could be designed that were retained in CY1 for at least 30 months in citrus without the detection of any deletion-containing variants. These findings demonstrate that RNA substructures have thermodynamic properties in coding and non-coding regions of +RNA viral genomes necessary for genome integrity and strongly suggest that incorporating similar properties into VIGS inserts can stabilize the inserted sequences, which opens up VIGS applications for long-lived trees and vines.

## RESULTS

### Identifying sites that can accommodate insertions in CY1

Converting CY1 into a VIGS vector first required identifying sites capable of accepting foreign sequences without adversely affecting important viral functions such as replication, translation, and movement. The CY1 secondary structure, which was previously mapped using a combination of SHAPE RNA structure probing and phylogenetic comparisons (20), subdivides into three domains (Fig. 1B). The 5ʹ terminal domain 1 (positions 1–668) contains the −1 programmed ribosomal frameshift (−1PRF) site and is separated from the 3ʹ terminal domain 3 (position 2399–2692) by a stem (labeled the “bridge”) that supports domain 2 (position 669–2398). To avoid disrupting the RdRp coding region (positions 9–2161), insert sites needed to be within the 571 nt 3ʹ UTR, which encompasses part of domain 2 (positions 2162–2398) and all of domain 3. Domain 3 contains elements just across the bridge and near the 3ʹ terminus that participate in −1PRF through long-distance RNA:RNA interactions (25). In addition, domain 3 harbors the eIF4G-binding, 3ʹ cap-independent translation enhancer (3ʹCITE) (24), as well as two hairpins near the 3ʹ terminus that are conserved in related umbraviruses and carmoviruses where they are required for replication (Fig. 1B) (2, 26). Considering this information, locations evaluated as possible insertion sites were mainly in single-stranded internal and apical loops in the 3ʹ UTR portion of domain 2.

**Fig 2:**
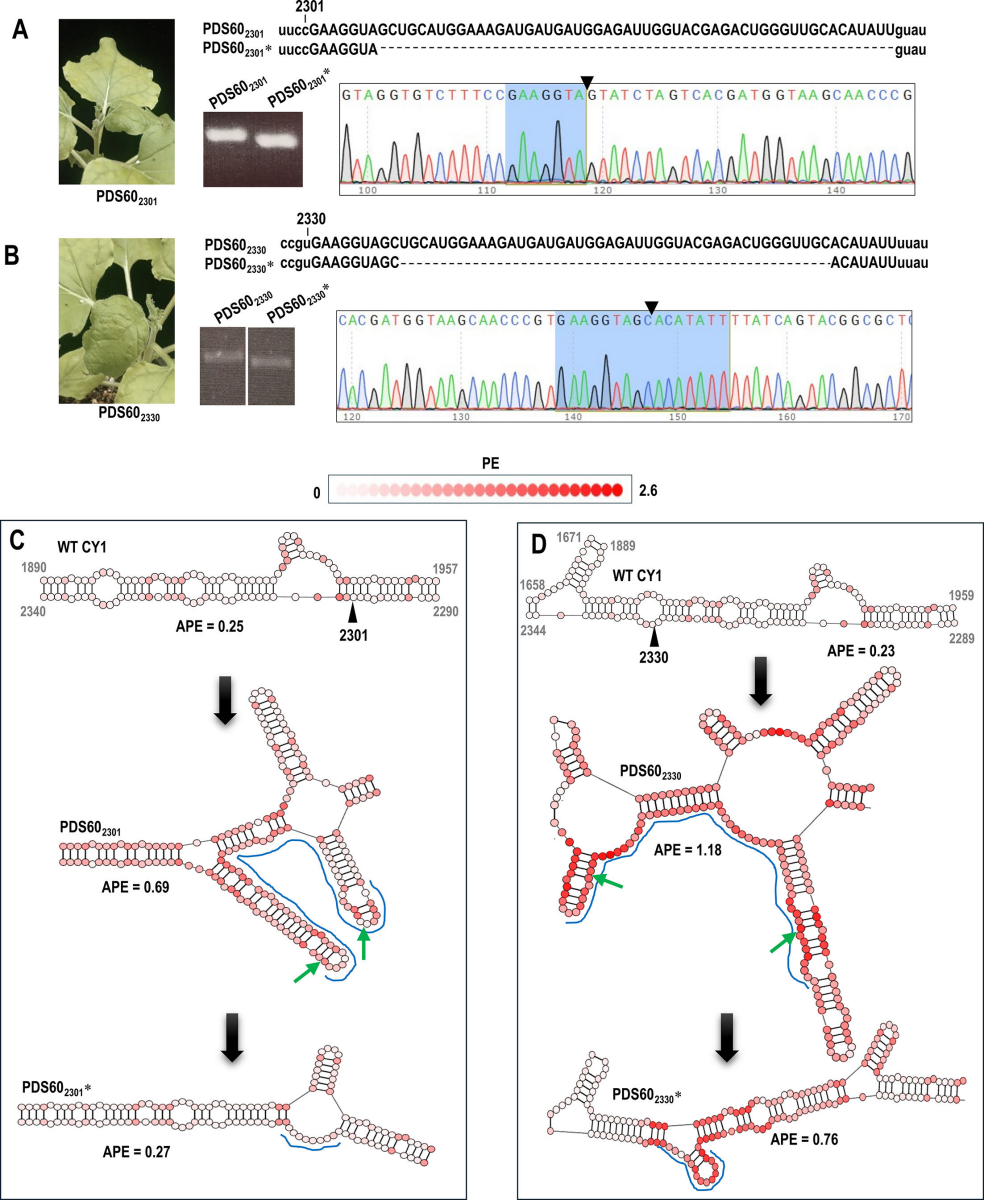
PDS60 inserted at either position 2301 or 2330 is not maintained. (**A**) Left, arrow points to symptomatic *N. benthamiana* leaf denoting infection by PDS60_2301_. Right, the inserted sequence is in upper case. PCR and RT-PCR of CY1 positions 2052–2489 using the parental construct or virus accumulating in infected plants, respectively. Left lane, PCR of parental CY1 PDS60_2301_. Right lane, RT-PCR of PDS60_2301_* extracted from systemic leaves at 3 wpi (‘*' denotes virus accumulating in infected plants to differentiate from the parental construct). A chromatogram was obtained from batch sequencing the faster migrating PDS60_2301_* product. The deletion point in the chromatogram is marked with an arrowhead. The remaining sequence of the insert after the deletion is highlighted in blue. (**B**) Same as A, but the construct contained PDS60 at position 2330. (**C**) Top, secondary structure of CY1 in the vicinity of the 2301 insert site. Middle, predicted structure with the PDS60 insert. Hatched line denotes the inserted sequence. Bottom, predicted structure after the deletion of most of the insert. Color coding reflects PE values for each nucleotide. Green arrows denote the deletion end points. (**D**) Same as C, but for PDS60 inserted at position 2330.

As the bridge is likely critical for the structural integrity of the viral genome, this region was avoided, and the positions selected were 2250, 2301, 2304, 2319, and 2330 within domains 2 and 2426 within domain 3 (note that 2301 was previously located in single-stranded interior loop in an earlier version of the structure [20] [Fig. 1B]). For positions 2250, 2301, 2319, 2330, and 2426, inserted sequences initially included a variety of segments from coding regions of different genes and ranged in size from 60 to 516 nt (Fig. S1). CY1 transcripts containing the inserts were first evaluated for p21 and p81 translational efficiency using wheat germ extract (WGE) and then for systemic movement in *N. benthamiana* plants. Full-length viral transcripts containing insertions in domain 2 generated similar levels of p21 and p81 as parental CY1, whereas sequences inserted at position 2426 within domain 3 significantly reduced the translation of both p21 and p81 (Fig. S1B). For evaluating infectivity, *Agrobacterium tumefaciens-*mediated vacuum infiltration was used to launch the viral constructs into *N. benthamiana*, as previously described (18). At 3 weeks post-infiltration (wpi), only plants infected with CY1 containing the shortest segment (PDS60; 60 nt) inserted into all domain 2 sites exhibited the typical CY1 early symptoms of stunting and cupping of a single apical leaf (Fig. 2A and B). These symptoms were not detectable by reverse transcription PCR (RT-PCR) of the negative plants (Fig. S1C). Based on an updated CY1 structure map for the region made possible with the availability of newly reported ULV sequences (Fig. 1B), the 2304 location was used to investigate the stability of hairpin structures that are described in the following sections.

### PDS60 inserts in CY1 increased RNA plasticity in the inserted region and were not retained

Inserts designed to target PDS mRNA were used because efficient VIGS activity causes photobleaching (7). However, none of the plants infected with CY1 carrying PDS60 exhibited the silenced phenotype (Fig. 2A and B). To examine whether the PDS60 inserts were maintained in the vectors, total RNA was extracted from plants infected with CY1 carrying PDS60 at positions 2301 (PDS60_2301_) or 2330 (PDS60_2330_) at 3 wpi and subjected to RT-PCR using primers specific for sequences that flanked the inserts. The only band discernible for each RNA sample migrated faster than the PCR product produced using the parental virus constructs as templates (Fig. 2A and B). Batch sequencing of the faster migrating RT-PCR products for PDS60_2301_* and PDS60_2330_* (asterisk denotes virus accumulating in infected plants) revealed the absence of 53 nt and 44 nt within the inserted sequences, respectively (Fig. 2A and B). These results indicated that, as with other VIGS vectors, sequences inserted into CY1 are not maintained.

The two key determinants of RNA plasticity are structural PE and minimal free energy (MFE), also known as Gibbs free energy (∆G) (27, 28). PE reflects the variety of states that a residue adopts in an RNA secondary structure (e.g., number of possible pairing partners and how often a residue is unpaired), and values can be obtained using the ViennaRNA folding software (see Materials and Methods). A residue has a PE value of 0 if the residue is always predicted to be unpaired or is always paired with the same partner residue, and PE values in this study ranged up to 2.6 for residues that were predicted to have the greatest number of alternative pairing states (27). ΔG values were also available through the same folding software, and a more negative ΔG value for similar RNA structures implies greater thermodynamic stability (lower plasticity) due to an increased number of hydrogen bonds and/or base stacking interactions (29, 30). As shown in Fig. 2C and D, the average PE (APE) in the region of the 2301 insert site is 0.25, and the APE for the nearby 2330 insertion site is 0.27. The addition of PDS60 at position 2301 is predicted to increase the regional APE to 0.69, which is reduced to 0.27 following the deletion. For position 2330, the addition of PDS60 increases the APE to 1.18, which was reduced to 0.76 after the deletion. These results suggest that a reduction in regional APE may be associated with enhanced fitness following partial insert deletion.

### Insertion of a low PE hairpin designed to target green fluorescent protein induced efficient silencing but was not maintained

**Fig 3:**
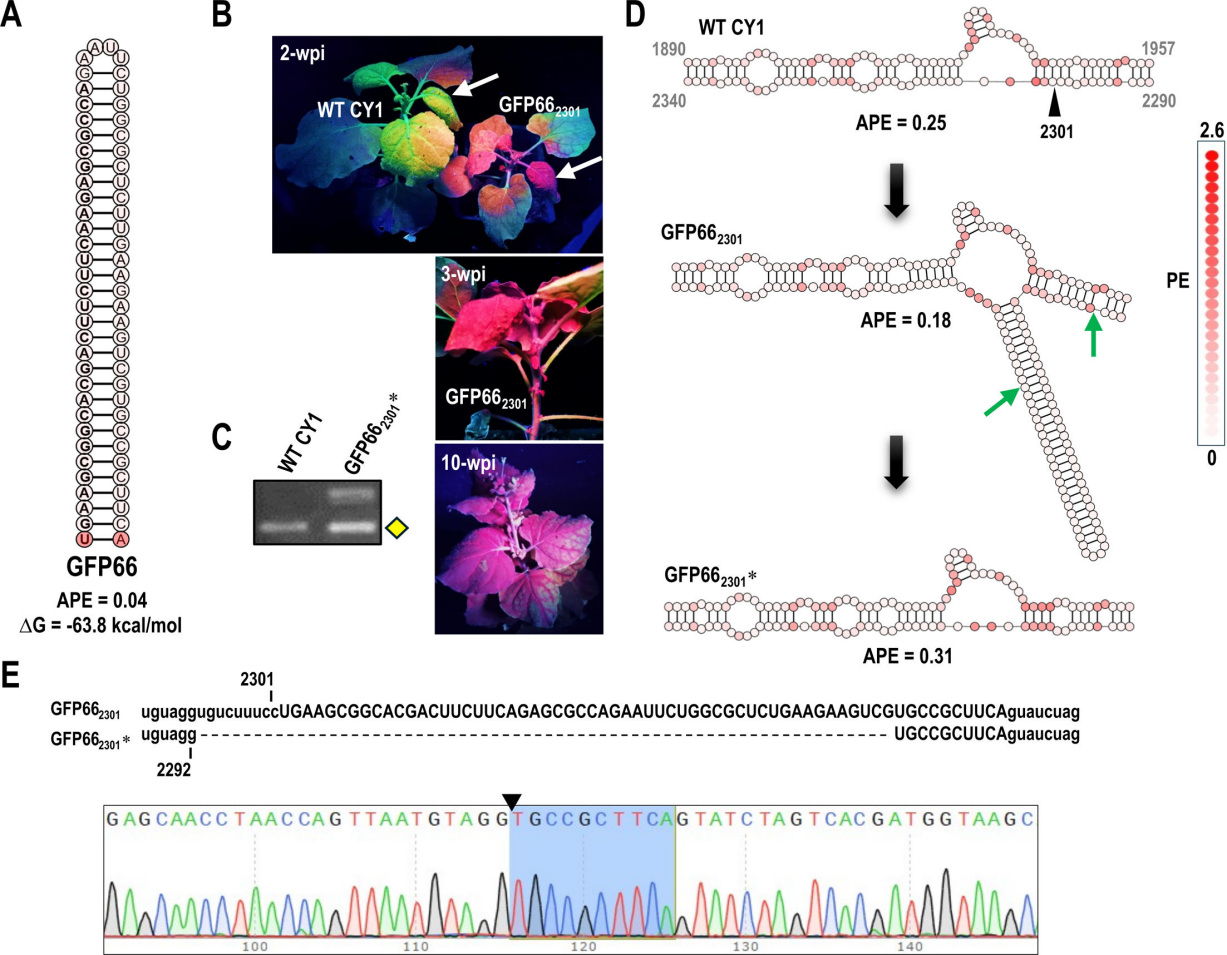
CY1 VIGS vector with hairpin insert at position 2301 silenced GFP in *N. benthamiana* 16c despite low insert retention. (**A**) Sequence and structure of the 66-nt hairpin targeting GFP that was inserted at position 2301 (construct GFP66_2301_). The GFP-targeting sequence is on the 5ʹ side of the hairpin. Color coding reflects PE values for each nucleotide. (**B**) GFP66_2301_ silencing of GFP. Normal plant tissue fluoresces red, which is masked by GFP expression in the transgenic plants. Arrows denote the single cupped leaf that is the first visible phenotypic change in infected plants. Upper panel, the initial stage of GFP silencing at 2 wpi. Middle panel, systemic spread of GFP silencing at 3 wpi. Lower panel, GFP silencing at 10 wpi. (**C**) GFP66 is poorly maintained in the vector at 3 wpi. RT-PCR products from amplifying positions 2052–2489 are shown. WT CY1, CY1-infected plants; GFP66_2301_*, GFP66_2301_ accumulating in systemic leaves. Yellow diamond denotes the deletion product subjected to batch sequencing in (**E**). (**D**) PE profile changes near the 2301 insertion site before and after the deletion. Green arrows denote the deletion end points. (**E**) Sequence alignment between parental GFP66_2301_ and batch sequence obtained for the lower RT-PCR band from GFP66_2301_* shown in (**C**). Sequencing chromatogram indicates that the PCR band was generated from a single major deletion species. The triangle denotes the deletion site. The remaining insert sequence after the deletion is highlighted in blue.

Simple, unbranched hairpins with low APE (i.e., low structural plasticity) are predicted to be structurally stable. As inserts, such hairpins should cause fewer structural alterations to the viral backbone at the insertion site compared with less structured sequences (e.g., PDS60). A fully base-paired 66-nt hairpin (GFP66) was therefore designed to target green fluorescent protein (GFP) mRNA expressed by transgenic *N. benthamiana* 16c, a well-established model for assessing VIGS efficacy (31) (Fig. 3A). GFP66, with its very low APE of 0.04, was inserted into position 2301 of CY1 (GFP66_2301_), which was not predicted to substantially change the structure or APE in the region (Fig. 3D). At 2 wpi, GFP silencing was observed in upper systemic leaves of several infiltrated plants, coinciding with a typical early cupped leaf (Fig. 3B, upper panel, arrow). Within 7 days of symptom onset, GFP silencing became more widespread (Fig. 3B, middle panel) and persisted until plant senescence (Fig. 3B, lower panel). RT-PCR analysis of GFP66_2301_* at 3 wpi revealed a mixed population of viruses in one infected plant, including a major deletion-containing variant whose RT-PCR product co-migrated with the PCR product of wild-type (WT) CY1 (Fig. 3C). Batch sequencing of this RT-PCR product revealed the loss of 9 nt of CY1 sequence and 57 nt of GFP66 (Fig. 3E), with no additional variants detectable in the sequencing chromatogram as indicated by the uniform peaks. These results indicate that CY1 can still serve as an effective VIGS vector for targeting host gene expression, despite the emergence of a subpopulation carrying a significant deletion during infection (Fig. 3E). However, even a fully base-paired hairpin with low APE was not maintained when inserted at position 2301, suggesting that low APE alone is insufficient for insert maintenance.

**Fig 4:**
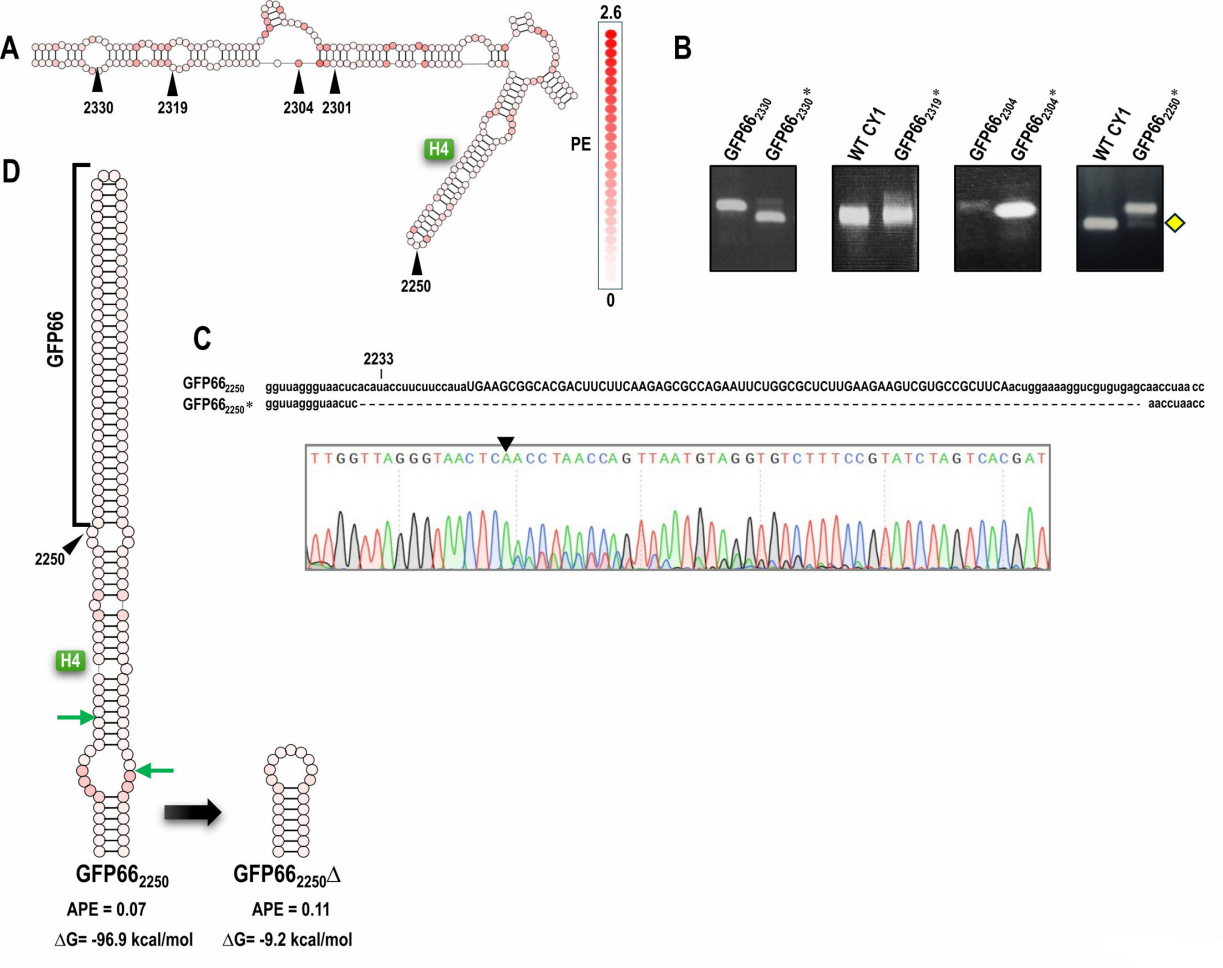
Screening other insertion sites for retention of GFP66. (**A**) Location of the five insert sites in CY1 that were used to screen retention of GFP66. Color coding reflects PE values for each nucleotide. (**B**) RT-PCR (positions 2052–2489) of CY1 containing GFP66 at the different insertion sites at 3 wpi. Left lanes are PCR products of either WT CY1 plasmid or parental construct plasmids. Yellow diamond denotes the deletion product subjected to batch sequencing. (**C**) Chromatogram from batch sequencing the lower PCR product from plants infected with GFP66_2250_. One major deletion variant was identified (shown), and other unidentified variants were present as visible by the non-singular nucleotide peaks on the sequencing chromatogram. (**D**) Predicted structure of the GFP66 insert at position 2250 in the apical loop of hairpin H4 and the major deletion variant that removed the insert and most of H4. Bracket denotes the GFP66 insert. Green arrows denote the deletion end points.

As mentioned above, CY1 structure near position 2301 was re-evaluated when additional class 2 ULV sequences became available, which suggested that position 2301 might be in a paired configuration. To determine if this site was not amenable to hairpin persistence, GFP66 was also inserted at positions 2250, 2304, 2319, and 2330 (Fig. 4A). RT-PCR analysis of progeny at 3 wpi revealed that GFP66 was not fully retained at any of these locations, indicating that the loss of GFP66 was not site specific (Fig. 4B). Interestingly, when GFP66 was inserted into the apical loop of natural hairpin H4 at position 2250, some progeny contained a deletion that removed the insert and the majority of the natural hairpin, leaving a small 23 nt hairpin in its place (Fig. 4C and D). This indicates that H4, which is structurally conserved in all related ULVs, is not required for CY1 infectivity, but it is possible that a hairpin at this location is important for fitness.

**Fig 5:**
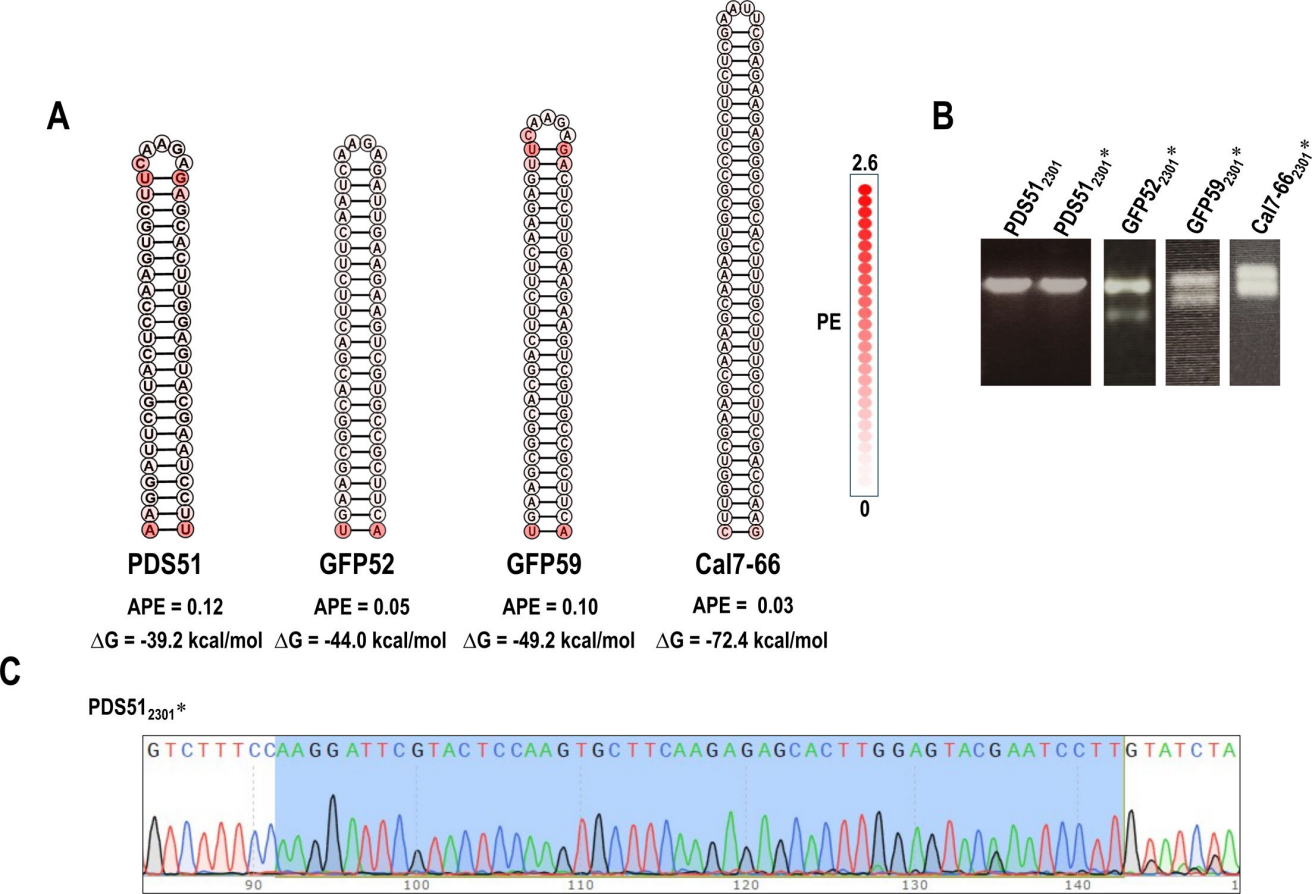
Retention of other fully base-paired hairpins inserted at position 2301. (**A**) Sequences and PE of the four hairpins that ranged in size from 51 to 66 nt. Color coding reflects PE values for each nucleotide. (**B**) RT-PCR (positions 2052–2489) of virus from infected plants at 3 wpi. The control at far left shows the PCR product for the PDS51_2301_ plasmid. (**C**) Batch sequencing of the RT-PCR product from PDS51_2301_* showing that the hairpin was maintained with no detectable alterations on the chromatogram. The retained sequence is marked in blue.

To determine if non-retention of GFP66 was due to its particular sequence, we evaluated four other fully base-paired, low APE hairpins for maintenance at position 2301 (Fig. 5A). As shown in Fig. 5B, hairpins with inserts of 52 nt, 59 nt, and 66 nt (GFP52, GFP59, and Cal7-66) were also not retained at 3 wpi. However, RNA extracted from plants infected with CY1 containing 51 nt PDS51 (PDS51_2301_*) generated a single RT-PCR product that co-migrated with the parental construct (Fig. 5B). Batch sequencing of the RT-PCR band revealed that the inserted hairpin was intact with no detectable variants at the sample collection time point (3 wpi; Fig. 5C). Both PDS51 and similarly-sized GFP52 had low APE, but they differed by 8.2% in their predicted ΔG values (−39.2 kcals/mol and −44.0 kcals/mol, respectively). This suggested the possibility that ΔG, the second key determinant of RNA plasticity, might be important for hairpin maintenance.

### CY1 natural hairpins are stable when duplicated and used as inserts

**Fig 6:**
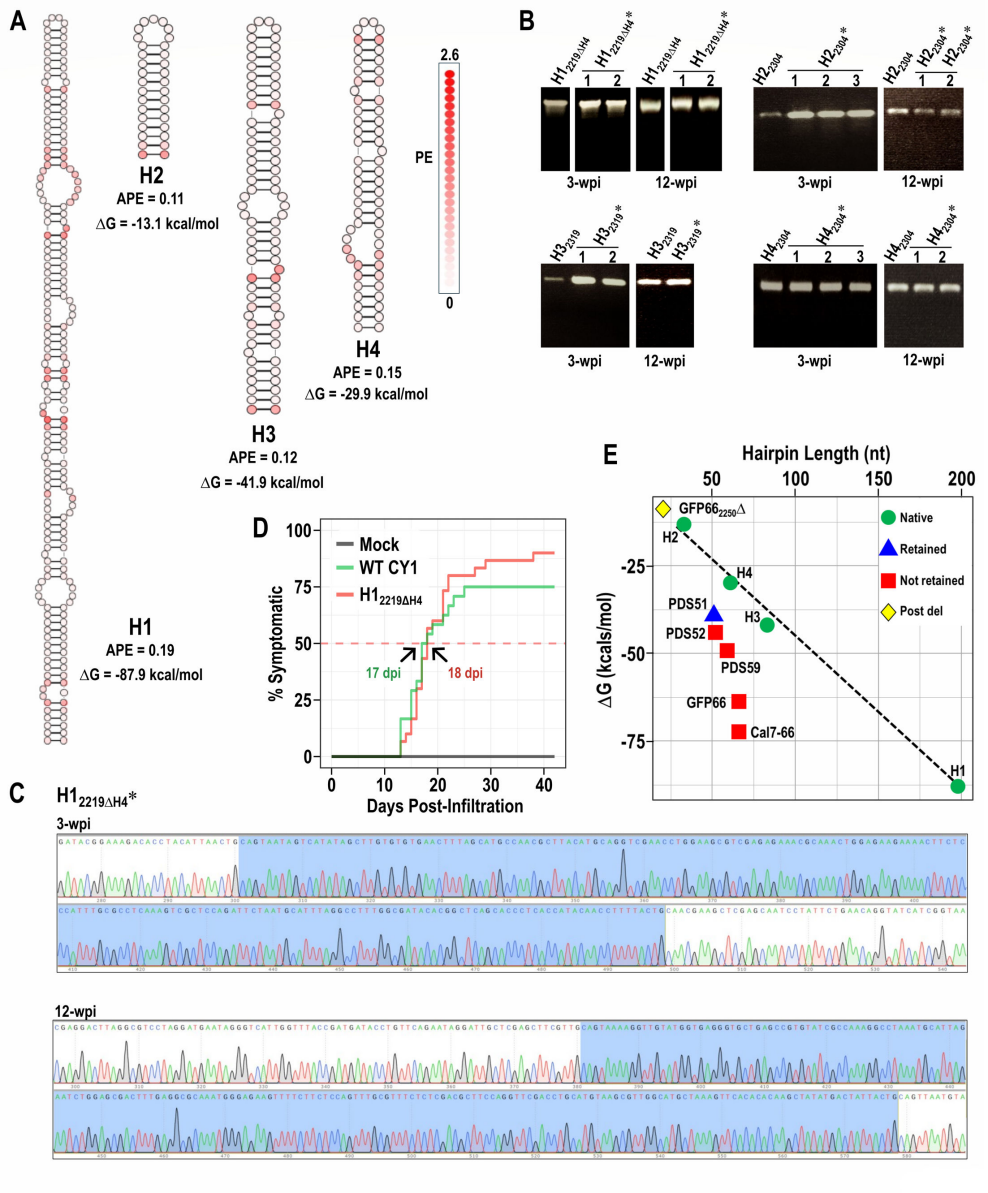
Retention of natural CY1 hairpins when duplicated and inserted into the CY1 genome. (**A**) PE, APE, and ΔG values for the four hairpins. See Fig. 1B for the natural location of the hairpins in the CY1 genome. Nucleotides are colored according to PE. (**B**) RT-PCR using RNA extracted from virus-infected plants at 3 and 12 wpi. Numbers reflect individual infected plants. Note that not all plants survived until 12 wpi. (**C**) Sequencing of H1_2219ΔH4_* RT-PCR product from one plant at 3 wpi and 12 wpi. The location of the insert is highlighted in blue. No variations from the parental sequences were discernible on the chromatograms. (**D**) Line graph showing the percent of symptomatic plants that were infiltrated with p19-only (Mock, dark gray), WT CY1 (green), or H1_2219ΔH4_ (pink). Plants were observed for symptoms daily over 6 weeks. (**E**) Graph showing the relationship between ΔG and hairpin length for retained and poorly retained hairpin inserts. Post del, hairpin formed from remaining viral sequences following the compared timing of symptom deletion of the insert.

Our hypothesis posits that viral genomes have evolved such that RNA substructures have precisely tuned thermodynamic properties that make the genomic RNA a fit template for replication and/or other functions. If correct, then natural CY1 hairpins in both coding and non-coding regions should already possess the appropriate thermodynamic properties, as non-persistence of at least coding-region hairpins would be lethal. To determine the validity of this hypothesis, four natural CY1 hairpins were selected for duplication and insertion into various domain 2 sites. These hairpins were H1 (197 nt), the longest unbranched hairpin; H2 (32 nt), the longest hairpin without internal loops; H3 (83 nt), the hairpin encompassing the p81 stop codon; and H4 (61 nt), the non-coding hairpin whose presence is not required (see Fig. 1B for locations within the genome; Fig. 6A). As predicted by Ancel and Fontana (15), H1, H2, H3, and H4 have low APE values (0.19, 0.11, 0.12, and 0.15, respectively) indicative of an evolutionary trend toward low plasticity. H1 and H3 were duplicated and inserted into the CY1 genome at position 2219, replacing H4 at this location, generating constructs H1_2219ΔH4_ and H3_2219ΔH4_. H2 and H4 were duplicated and inserted at position 2304, generating H2_2304_ and H4_2304_. As shown in Fig. 6B, RT-PCR products using RNA from symptomatic plants at 3 and 13 wpi comprised only single visible bands for all viral constructs, with the bands co-migrating with the PCR products of the parental constructs. Batch sequencing of the RT-PCR product of the largest insert, H1_2219ΔH4_*, at 3 and 13 wpi revealed no discernible changes to the inserted sequence (Fig. 6C). To confirm this result, the H1_2219ΔH4_*RT-PCR band at 13 wpi was cloned, and 11 clones were sequenced. All clones retained the identical sequence as the parental construct. This result strongly suggests that hairpins inserted into the CY1 genome might be retained if they contain the conformational and/or thermodynamic properties of evolved, natural hairpins.

To determine if the maintenance of additional sequence was detrimental to viral systemic infection, we compared the timing of symptom onset between WT CY1 and H1_2219ΔH4_ over 6 weeks (Fig. 6D). We found that plants infected with either virus began to display symptoms at similar times post-infiltration, reaching 50% symptomatic at a similar rate and produced a similar overall infection rate. This suggests that H1_2219ΔH4_ replicated similarly as WT CY1, despite containing an additional 138 nt (5% of the total viral genome).

To begin deciphering the properties of natural hairpins, we examined the relationship between hairpin ΔG and hairpin length. As shown in Fig. 6E, a linear correlation was found between the ΔG values of the natural CY1 hairpins and the length of the hairpin sequences. Since ΔG values are related to the number of base pairs in a structure, it was expected that longer, paired structures would possess a lower ΔG. Artificial hairpins that were not retained (GFP52, GFP59, GFP66, and Cal7-66) had ΔG values/hairpin length that were more negative (i.e., the artificial hairpins were more thermodynamically stable) than the natural hairpins primarily due to the higher number of base pairs. In contrast, the small hairpin remaining following the deletion of GFP66 that was inserted at position 2250 (GFP66_2250_Δ) had a ΔG value in line with those of the natural hairpins. Since the ΔG value of well-retained hairpin PDS51 was only predicted to be 8.2% higher than poorly retained GFP52, this suggests that if ΔG is important, even minor differences may impact maintenance, or the predicted ΔG value for this hairpin may not be correct due to the inserted sequence adopting a conformation that differs from the one predicted.

### GFP59 and GFP66 altered to mimic the structure and thermodynamic properties of natural hairpin H4 were maintained in the CY1 vector

**Fig 7:**
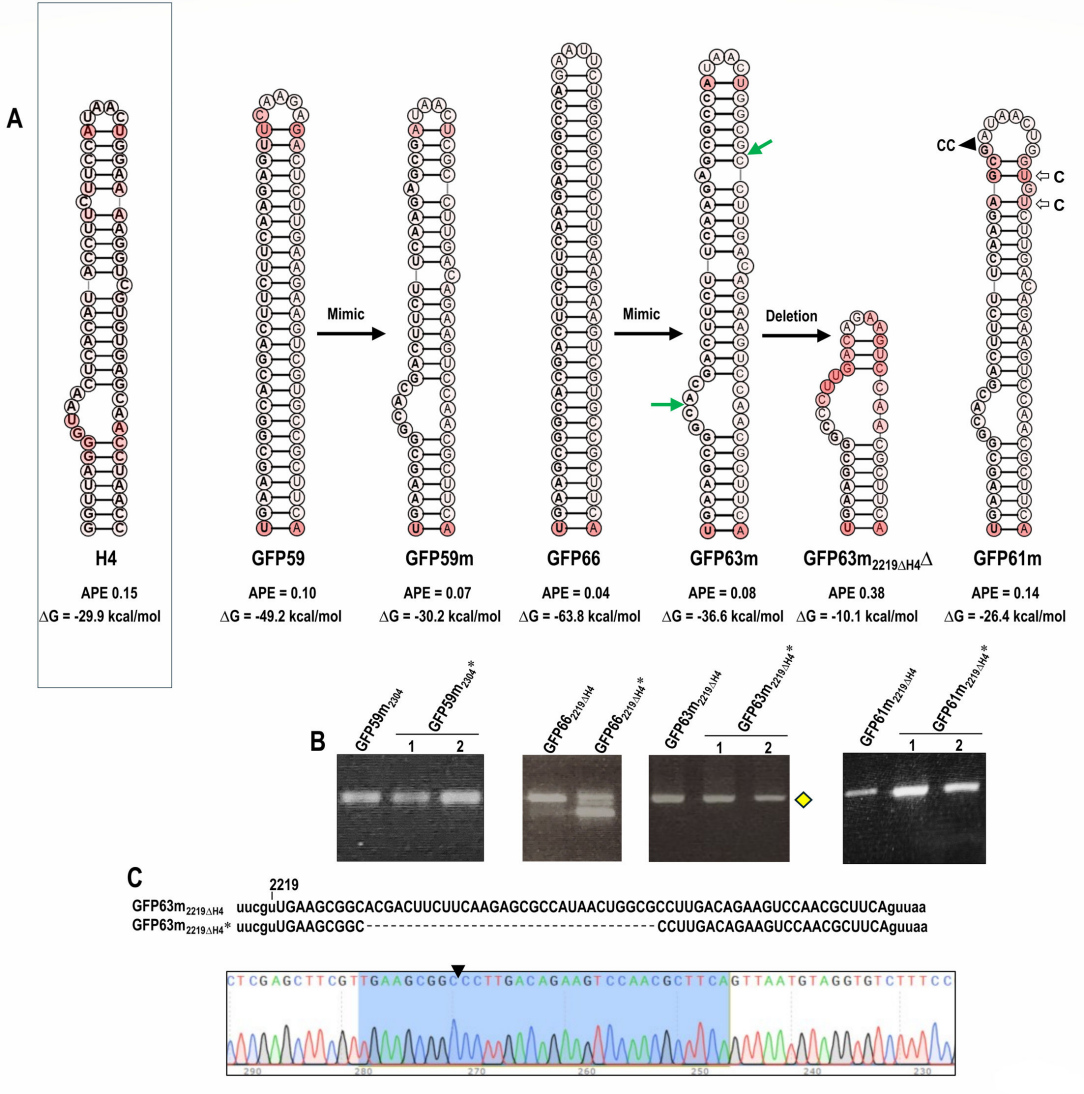
Stabilization of GFP59 and GFP66 by mimicking the structure and ΔG of natural CY1 hairpin H4. (**A**) Sequences and secondary structures of H4 and the mimicked hairpins. GFP59m, sequences on the 3ʹ side of non-retained hairpin GFP59 were modified to structurally resemble H4 with a similar ΔG, and hairpin was inserted at position 2304; GFP63m, sequences on the 3ʹ side of non-retained hairpin GFP66 were modified to structurally resemble H4 but with a lower ΔG, with hairpin inserted at position 2219 with a deletion of H4 (2219ΔH4); GFP63m_2219ΔH4_Δ, deletion product of poorly maintained GFP63m at position 2219ΔH4 (marked by yellow diamond in B); GFP61m: additional modifications that were made in GFP63m to increase the ΔG are shown. Green arrows denote deletion end points. (**B**) PCR products of the parental plasmids or WT CY1 plasmid and RT-PCR using RNA from infected plants. Yellow diamond denotes the RT-PCR product that was revealed to have a deletion when batch sequenced (see C). Non-retained hairpin GFP66 was also assayed at position 2219ΔH4. Numbers represent individual infected plants. (**C**) Sequencing of RT-PCR product from plants infected with GFP66_2219ΔH4_*. Chromatogram shows a single deletion variant. The remaining sequence of the inserted nucleotide after deletion is marked in blue. The triangle denotes the deletion site.

The retention of all four natural hairpins when duplicated and inserted into domain 2 locations suggested that designing well-retained hairpins should be possible if they are engineered with properties conforming to those of natural CY1 hairpins. To understand the importance of natural hairpin conformation and/or ΔG, we altered the conformations of poorly retained hairpins GFP59 (59 nt) and GFP66 (66 nt) to mimic those of similarly sized H4 (61 nt) by altering only the 3ʹ side, as 5ʹ side sequences were originally intended to target GFP gene expression. For GFP59, the 5 nt apical loop was replaced with the H4 tetraloop (UAAC), and internal loops and bulges were incorporated that were similar to those found in H4 (Fig. 7A). The resultant hairpin, GFP59m, maintained the low APE of GFP59, while the ΔG was increased from −49.2 kcals/mol (GFP59) to −30.2 kcals/mol (GFP59m), a value similar to that of H4 (−29.9 kcals/mol). GFP59m was inserted at position 2304 in the CY1 genome. At 3 wpi, GFP59m_2304_* generated a single discernible RT-PCR fragment that co-migrated with parental GFP59m_2304_, and batch sequencing the fragment revealed that the inserted hairpin was intact (Fig. S2).

For GFP66, similar alterations to the 3ʹ side changed its conformation to resemble that of H4, but these alterations were chosen to generate a hairpin that was more thermodynamically stable than H4, with a ΔG of −36 kcals/mol (20% more negative than H4). This hairpin, GFP63m, was inserted at position 2219 after the deletion of H4, generating GFP63m_2219ΔH4_. GFP66, which had not previously been evaluated at position 2219ΔH4, was not fully maintained at this location as with the other domain 2 locations (Fig. 7B). GFP63m initially appeared to be maintained at position 2219ΔH4, as shown by RNA from infected plants generating a single RT-PCR fragment at 3 wpi that seemed to co-migrate with the parental construct. However, batch sequencing the RT-PCR product for GFP63m_2219ΔH4_* revealed that only 33 nt was retained (Fig. 7C), with the remaining sequence predicted to adopt a hairpin structure (Fig. 7A, GFP63m_2219ΔH4_Δ). To evaluate if the lower ΔG of GFP63m contributed to the lack of hairpin retention, GFP63m was further modified by deleting two cytidylates and converting two cytidylates to uridylates (GFP61m), reducing the ΔG to −26.4 kcals/mol. Unlike GFP63m, GFP61m was well maintained when inserted into position 2219ΔH4 (Fig. 7B), as confirmed by batch sequencing (Fig. S2). These findings suggest that mimicking the structure of a natural CY1 hairpin can result in the retention of foreign sequences if the ΔG/hairpin length is similar to, or higher, than that of the natural hairpin.

### Similar-sized hairpins designed to be retained or not retained matched their predicted phenotype in the CY1 vector

**Fig 8:**
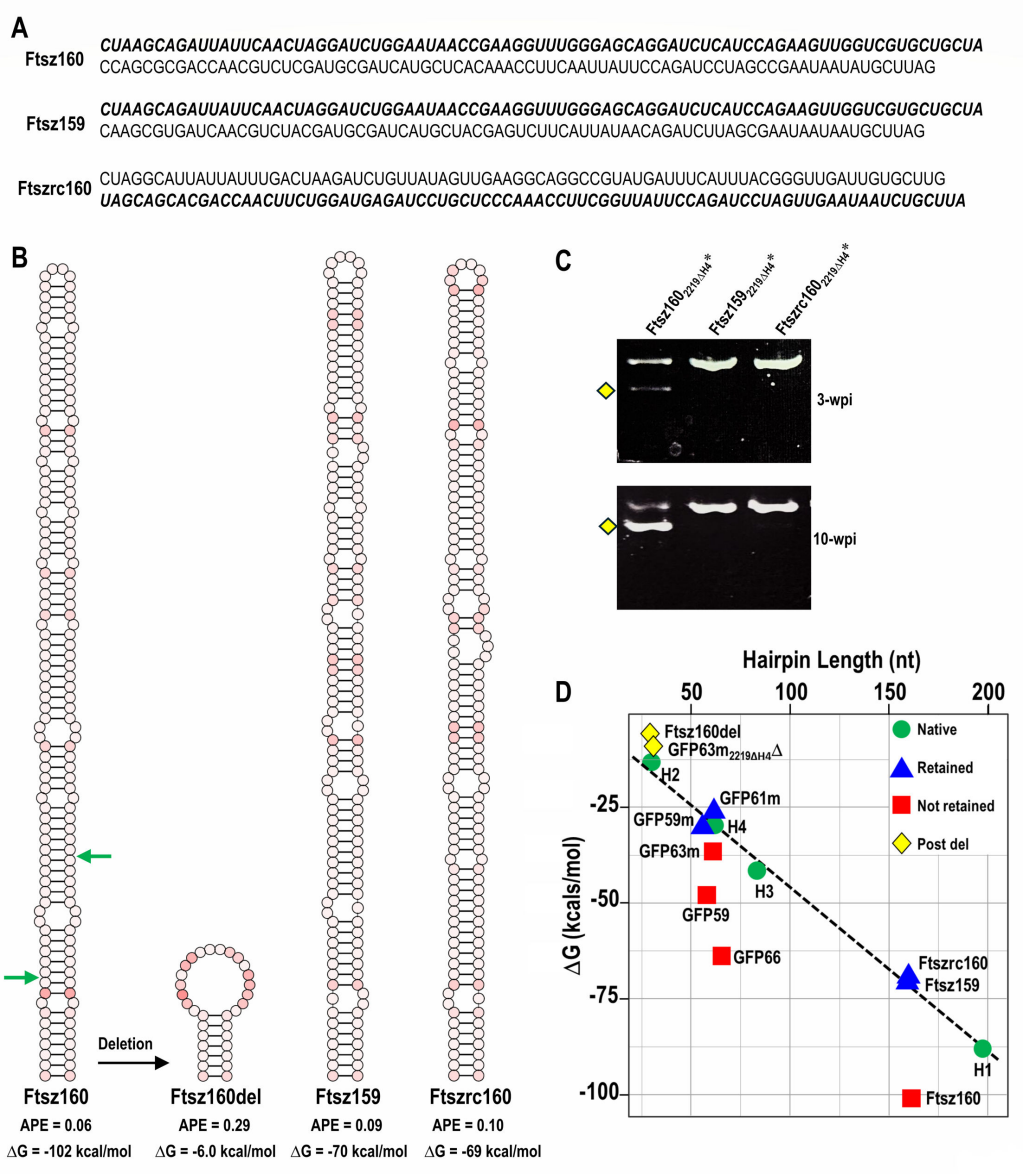
Design of hairpins with appropriate and inappropriate ΔGs. (**A**) Sequences of Ftsz160, Ftsz159, and Ftszrc160. Bold sequences are identical in Ftsz160 and Ftsz159 and are the reverse complement of the sequence in Ftszrc160. (**B**) Secondary structures of the three hairpins and a deletion product derived in plants from Ftsz160 (marked by yellow diamond in C). The three designed hairpins were inserted at position 2219ΔH4 in CY1. Green arrows denote deletion end points. (**C**) RT-PCR products of Ftsz160_2219ΔH4_*, Ftsz159_2219ΔH4_*, and Ftszrc160_2219ΔH4_* (see Fig. S3 for batch sequencing). (**D**) Graph showing the relationship between ΔG and hairpin length for retained and poorly retained parental, mimic, and designed hairpin inserts. Post del, hairpin formed from remaining viral sequences following deletion in the insert.

The linear correlation between hairpin lengths and ΔG values for the natural CY1 hairpins, coupled with the finding that raising the ΔG of mimic hairpin GFP63m allowed for maintenance in the CY1 vector, suggested that hairpin inserts could be designed for retention if they contained the appropriate ΔG, irrespective of whether or not their conformations mimicked those of natural hairpins. Based on the slope of the line in the graph shown in Fig. 6E, the corresponding ΔG value (*y*) for a hairpin of size *x* can be calculated (*y* = −0.42*x* – 2.84, *R*^2^ = 0.988, *F* (1, 2) = 237.7, *P* = 0.004). Using this calculation, a hairpin of 160 nt should be retained in CY1 if it has a ΔG of ~70 kcals/mol (95% CI: −92.4 to −52.4 kcals/mol). To test this prediction, three new hairpins (159–160 nt) were designed with similar overall conformations unrelated to those of the natural hairpins. Two of the hairpins contained ΔG’s that conformed to those of the natural hairpins: Ftsz159 (ΔG = −70 kcals/mol) and Ftszrc160 (ΔG = −69 kcals/mol). The third hairpin (Ftsz160) was designed to have a ΔG that was more negative (ΔG = −102 kcals/mol; Fig. 8A and B). Ftsz159 and Ftsz160 had identical sequences on their 5ʹ sides, whereas the 5ʹ side of Ftszrc160 contained the reverse complement of this sequence.

All three hairpins were inserted at CY1 position 2219ΔH4. At 3 and 10 wpi, RT-PCR products of Ftsz159_2219ΔΗ4_* and Ftszrc160_2219ΔΗ4_*generated single bands the size of the parental constructs, indicating that these hairpins were well maintained. Batch sequencing of the 10 wpi RT-PCR products confirmed that there were no deletions or base alterations in these hairpins (Fig. S3). In contrast, CY1 containing Ftsz160 generated two RT-PCR products, with the lower band containing only 32 nt of the original hairpin (Fig. 8B; Fig. S3). These data, together with the data for the mimic constructs, support the importance of ΔG/length in designing hairpins that are well maintained in the CY1 VIGS vector (Fig. 8D).

### Both ΔG and PE distribution are important for hairpin maintenance in CY1

**Fig 9:**
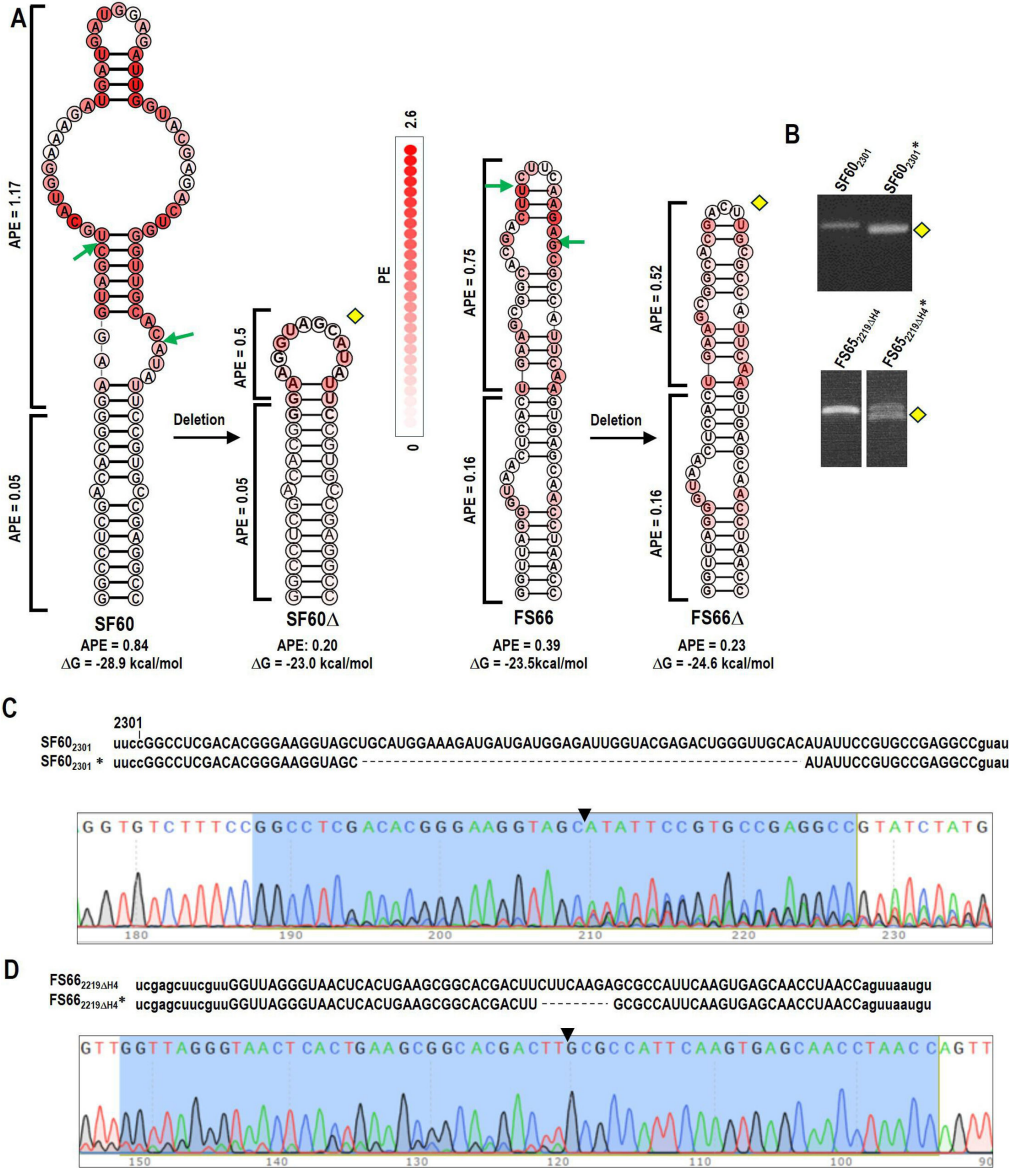
Hairpins designed with appropriate ΔG but high regional PE values were not maintained. (**A**) Secondary structure and thermodynamic properties of SF60, FS66, and their deletion variants (denoted by yellow diamonds). SF60 was inserted into position 2301, and FS66 was inserted into 2219ΔH4. Green arrows denote deletion end points. (**B**) RT-PCR of RNA from plants at 3 wpi with SF60_2301_ and FS66_2219ΔH4_. Yellow diamond denotes the deletion product subjected to batch sequencing. (**C**) Sequence alignment of SF60_2301_* with its parental construct and chromatogram from batch sequencing the RT-PCR product. Note heterogeneity indicating that the deletion variant is not the only virus accumulating in the plants. The remaining insert sequences after deletion are marked in blue. The triangle marks the location of the deletion. (**D**) Same as C but for FS66_2219ΔΗ4_*.

Since all hairpins assayed above had low APE values (0.19 or lower), whether or not they were well maintained, a question remained as to the importance of low APE for hairpin design. To test if residue PE affects hairpin maintenance, hairpins SF60 and FS66 were designed with appropriate ΔG values but with residues containing substantially higher PE values in their apical regions (Fig. 9A). SF60 contained an artificially designed base region with low APE (0.05), and a 60 nt upper region with a high APE (1.17). FS66 contained the 31-nt lower region of H4 (APE = 0.16) with a 35 nt upper region designed to a have high APE (0.75). SF60 was inserted at position 2301, and FS66 was inserted at 2219ΔH4. At 3 wpi, RT-PCR of the RNA from infected plants revealed that neither hairpin was maintained (Fig. 9B). Sequencing the deletion RT-PCR products revealed truncations in the high APE region that reduced apical APE values. These data support the importance of low APE throughout a hairpin as well as appropriate ΔG for maintenance of hairpin inserts in CY1.

### Designed hairpin with appropriate thermodynamic properties is retained in CY1 for at least 30 months in citrus

**Fig 10:**
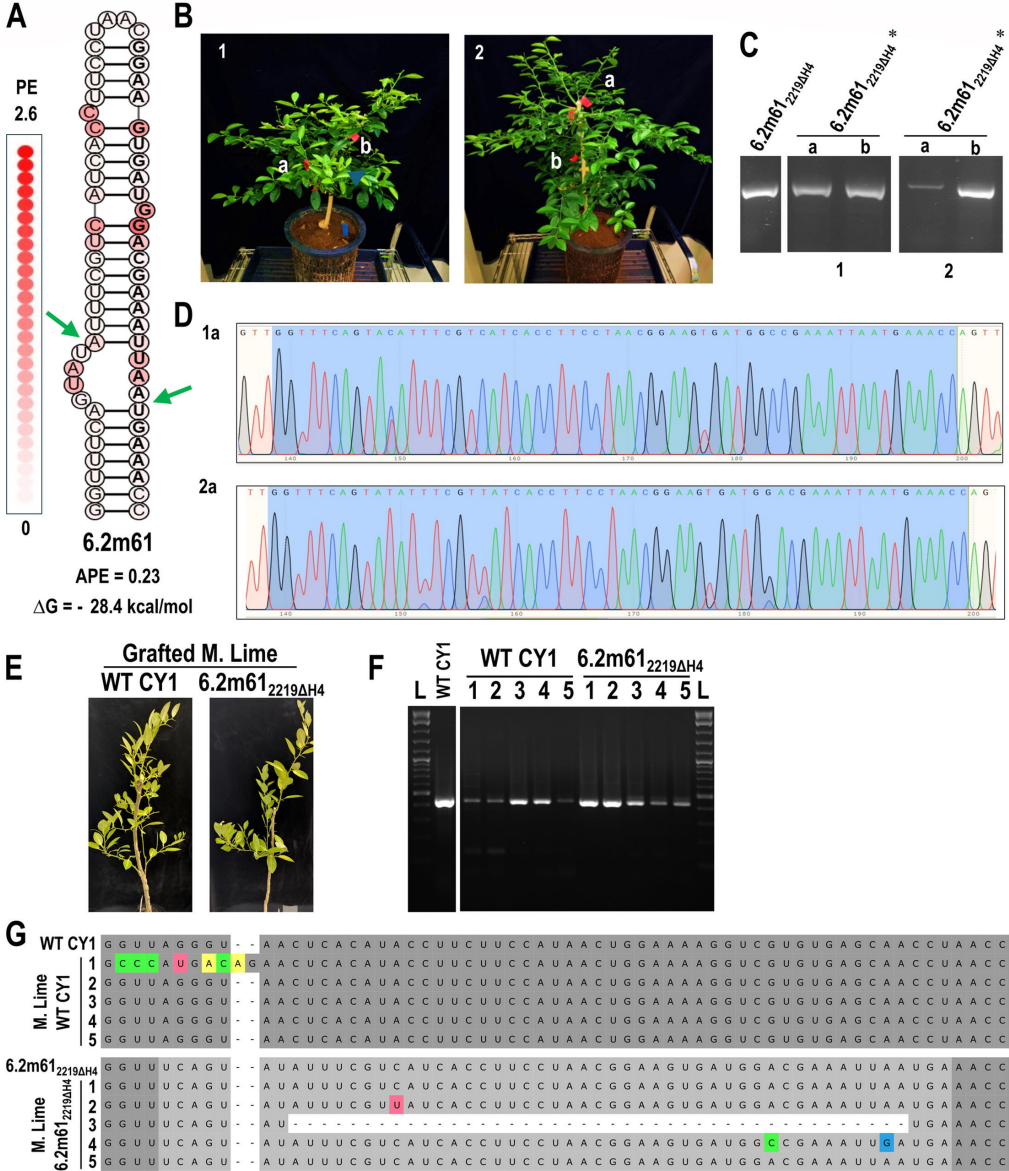
Low APE hairpin with appropriate ΔG is retained for at least 30 months. (**A**) Secondary structure of 6.2m61, an H4 mimic construct inserted into the 2219ΔH4 site. Green arrows denote the deletion end points. (**B**) Mexican lime plants at 30 months post-infiltration (mpi). (**C**) RT-PCR of 6.2m61_2219ΔH4_* from two leaves in each of two plants at 30 mpi. (**D**) Chromatograms from batch sequencing RT-PCR products from 1a and 2a with the inserted sequence highlighted in blue. (**E**) Representative image (*n* = 5) of Mexican lime trees infected with WT CY1 or 6.2m61_2219ΔH4_ at 12 months post-grafting from plants shown in (**B**). (**F**) RT-PCR of RNA collected from five WT CY1- or 6.2m61_2219ΔH4_-infected trees. (**G**) Alignments of batch sequencing of PCR products from (**F**) with either WT CY1 or 6.2m61_2219ΔH4_. WT CY1 sequence is shown in dark gray, 6.2m61_2219ΔH4_ insert sequence is in light gray, and point mutations are colored based on nucleotide base.

The retention of hairpins with appropriate thermodynamic properties for at least 13 wpi in *N. benthamiana* demonstrated the potential for long-term retention of inserted hairpins for VIGS field applications. To evaluate if designed hairpins can be maintained for the longer times that are required for infection of a long-lived plant, a 61 nt hairpin designed as an H4 mimic (6.2m61) with ∆G = −28.4 kcals/mol and APE of 0.23 (Fig. 10A) was inserted into position 2219ΔH4, and the resultant construct was infiltrated into 1-year old *Citrus aurantifolia* (Mexican lime) trees. At 30 months post-infiltration, RNA samples were collected from two locations in two trees (Fig. 10B) and subjected to RT-PCR (unlike *N. benthamiana*, CY1 is symptomless in citrus [18]). The amplified products co-migrated with those of the parental construct (Fig. 10C), and chromatograms from batch sequencing the RT-PCR products revealed no detectable variants (Fig. 10D). To determine if the inserted hairpin was also stable after passaging into new plants, scions from WT CY1- and 6.2m61_2219ΔH4_-infected trees were grafted onto five new Mexican lime trees and tested at 12 months post-grafting. Once again, we observed neither symptoms nor differences in growth (Fig. 10E). RT-PCR of the region containing the insert confirmed the presence of CY1 in all plants (Fig. 10F). Batch sequencing of the RT-PCR products found high sequence conservation for WT CY1 and 6.2m61_2219ΔH4_ (Fig. 10G). Four of the five trees maintained the 6.2m61 insert for the combined infection time of 42 months, whereas one tree contained a virus with a deletion of most of the insert, leaving behind a small hairpin in the previous H4 location similar to the deletion product of GFP66_2250_ (Fig. 4D). This suggests that additional parameters may need to be explored (e.g., elimination of large internal bulges) to ensure even greater maintenance over extended time. Altogether, these results support our hypothesis that hairpin substructures can be maintained for long periods of time in +RNA viral genomes if they contain thermodynamic properties that match those of native hairpins, opening up VIGS applications for use in long-lived field crops.

## DISCUSSION

Although VIGS has been used for studying plant functional genomics for several decades, agricultural applications, particularly in long-lived trees and vines, have been significantly hampered by the instability of sequences inserted into the viral genome. As with many other VIGS vectors in which instability of foreign sequences has been reported (9, 11–13), initial insertions of short sequences (60 nt) and simple hairpins into multiple single-stranded sites within the CY1 3ʹ UTR were not tolerated with one exception (Fig. S1; Fig. 2 to 5). To determine why hairpin PDS51 was surprisingly maintained at 3 wpi, we formulated a hypothesis, based on theoretical work by Ancel and Fontana (15), that RNA viral genomes have evolved to reduce plasticity, which minimizes fitness costs that can impair functional efficiency. Since plasticity is a function of nucleotide PE and ΔG, we proposed that simple hairpin inserts will be retained if they contain thermodynamic properties that are compatible with a plant virus genome that has evolved to have the most advantageous conformation for its infection cycle.

The poor retention of low APE hairpins GFP66, GFP52, GFP59, and Cal7-66, in contrast to the less thermodynamically stable PDS51, suggested that a CY1 VIGS vector fitness may require that hairpin inserts possess particular thermodynamic properties that are similar to those of natural viral substructures, including appropriate ΔG values. The long-term retention of four natural CY1 hairpins when used as inserts, including hairpin H1 that is 197 nt long (Fig. 6), is consistent with this hypothesis, as was finding a linear relationship (*y* = −0.42*x* – 2.84) between ΔG and hairpin length. Also consistent with this hypothesis was the finding that non-maintained hairpins GFP66 and GFP59, whose ΔG values were lower than those of natural hairpins, could be retained for at least 3 wpi in CY1 if altered to mimic the conformation and ΔG/length of the similarly sized natural hairpin H4 (Fig. 7). A precisely mimicked conformation, however, was not necessary, as 160 nt hairpins were also retained when designed to have appropriate ΔG/length through a combination of unpaired and paired regions while maintaining low residue PE (Fig. 8).

The importance of maintaining low APE for insert retention was suggested by two findings. First, RNAfold web server-based analysis predicted that insertion of the low-structured fragment PDS60 at position 2301 or 2330 significantly elevated the APE near the insertion sites, which was accompanied by conformational changes in secondary structure (Fig. 2C and D). The deletions that occurred during 3 weeks following infiltration were predicted to reduce both the local APE and generate conformations more closely resembling the original structure in the region (Fig. 2C and D). Second, the insertion of hairpins SF60 and FS66 with high APEs in their apical regions led to the selective deletion of the high PE clusters (Fig. 9). All together, these results support our hypothesis that if thermodynamic features corresponding to natural CY1 RNA substructures are incorporated into hairpins inserted into locations that do not disrupt important *cis* elements, they will likely be maintained by the VIGS vector.

An analysis of the CY1 genome secondary structure suggests that other parameters might also be important for hairpin insert design. CY1 substructures have relatively short consecutive base-paired stretches, with the longest being the 13 base pairs of hairpin H2. Additionally, only six regions in the CY1 genome (positions 485–499, 786–806, 1396–1414, 1744–1756, 2072–2083, and 2826–2842) contain more than 10 consecutive non-Watson-Crick paired residues, the longest of which spans 21 nt (positions 786–806). The overall architecture of the CY1 genome suggests a continuous alternation between short base-paired stems and short unpaired regions (i.e., bulges, internal loops, and apical loops). Although the current secondary structure map does not account for the majority of tertiary interactions, it is likely that the genome maintains a moderate level of structural plasticity—neither too high nor too low—achieved through a balance of base-paired and unpaired regions. Since DCL endonucleases cleave dsRNA, it is also possible that these interspersed regions reduce recognition by antiviral defense mechanisms, which target fully double-stranded RNA (3, 4). Conversely, large regions of single-stranded RNA may be inherently less stable and provide a better target for the RISC complex. Under this model, the trends we have observed in the thermodynamics of CY1 hairpins may not directly contribute to stability but instead may describe properties of hairpins that are resistant to host antiviral mechanisms.

**Fig 11:**
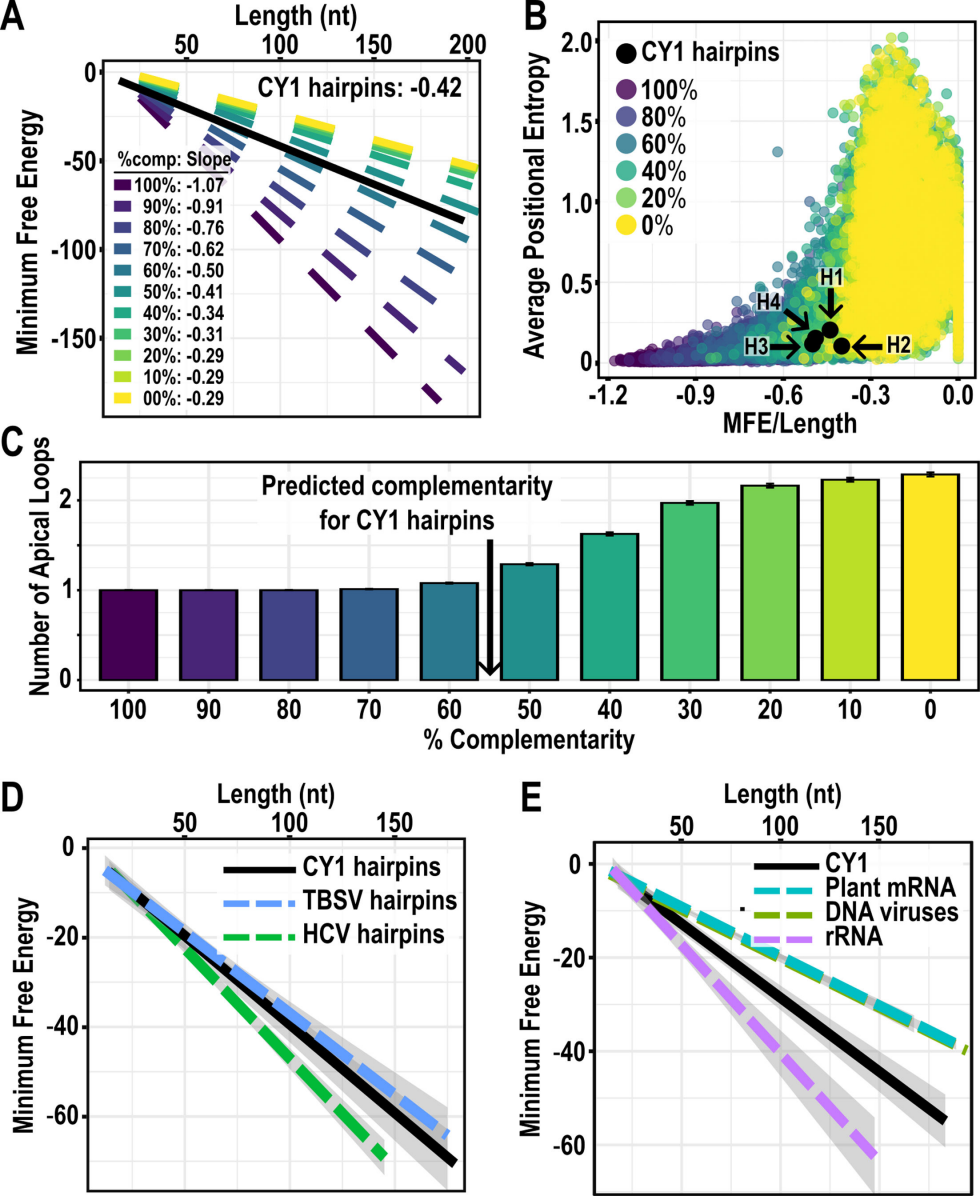
Analysis of thermodynamic characteristics of randomly generated hairpins. (**A**) The relationship between ΔG and the length of 38,577 random hairpins of various lengths and complementarity. Slopes for each complementarity set are shown and graphed along with the line generated by CY1 hairpins (black). (**B**) Graph comparing between ΔG/length and the average PE of the random hairpins. The four CY1 hairpins used as natural insertions are shown in black. (**C**) The number of apical loops present in the predicted folding of the randomly generated hairpins. Based on the slope found for CY1 hairpins in (**A**), the estimated position for CY1 hairpins is indicated. Error bars represent SE of 3,500 hairpins. (**D**) The relationship between ΔG and length of CY1 hairpins (black), tomato bushy stunt virus (TBSV) hairpins (blue), and hepatitis C virus (HCV) hairpins (green) from solved secondary structures. (**E**) The relationship between ΔG and length of CY1 hairpins (black) and human ribosomal RNA (purple) from solved structures, along with predicted hairpins found in RNA transcribed from DNA viruses (green) and plant mRNA (teal). Shaded areas for (**D**) and (**E**) represent SE of the linear regression.

By randomly generating 38,577 hairpins of varying lengths, nucleic acid compositions, and complementarity, we confirmed that the relationship between ΔG and hairpin length is mostly driven by the number of base pairs within a hairpin (Fig. 11A). Despite requiring particular secondary structures for viral functions, CY1 hairpins most closely resemble the slopes for randomly generated hairpins with only 50%–60% complementarity. This further supports a relationship between ΔG and length for CY1 that is a direct consequence of the balance between paired and unpaired regions. This balance becomes more apparent by graphing the ΔG/length ratio by the APE of the randomly generated hairpins, where we observed a logarithmic relationship between the two variables such that APE rapidly increases as ΔG/length increases (Fig. 11B). Importantly, natural CY1 hairpins H1–H4 are positioned to possess the highest ΔG/length ratio possible before APE rapidly increases. Indeed, the hairpin structures of randomly generated hairpins quickly break down when their complementarity is less than 50%, as indicated by the increasing number of apical loops predicted in these structures (Fig. 11C). Based on the ΔG/length relationship, CY1 hairpins possess as little complementarity (high ΔG) as possible while still reliably forming a simple hairpin.

This relationship between hairpin ΔG/length found for CY1 may also apply to other +RNA viruses but not to structures of large RNAs in general. An analysis of simple hairpins found in the full-length structures determined for tomato bushy stunt virus (TBSV) (23) and hepatitis C virus (HCV) (21) produced very similar relationships between ΔG and hairpin length as found for CY1 (Fig. 11D). In contrast, hairpins found in 16S and 28S ribosomal RNA produced a steeper slope, suggesting a higher percentage of high complementary hairpins and more stable structures. In contrast, hairpins predicted by *in silico* folding of plant mRNAs, as well as transcripts from plant and animal DNA viruses, produced less steep slopes similar to that of unstructured randomly generated hairpins (<30% complementarity [Fig. 11E]). Together, these observations suggest that +RNA viruses have evolved RNA structures that fit between the extremely structured ribosomal RNA and the much less structured mRNA from both plants and DNA viruses.

One possibility for why inserts are rapidly deleted if they contain unacceptable thermodynamic properties (high APE and low ΔG/length) is that these properties create RdRp recombination hotspots within the CY1 genome. Recombination hotspots have been widely studied in many RNA viruses and are often found adjacent to stem-loop structures (32–34) or within unstructured regions with low base-pairing probability (34, 35). Regions of low ΔG/low plasticity may cause polymerase pausing, promoting the premature release of the RNA template. If the RdRp maintains its association with the truncated nascent strand, it can resume transcription at a second location on the same or a different template (36–39). If the restart site is close to the initial release site, a local deletion occurs, aligning with our observations for GFP52, GFP59, GFP66, and Cal7-66 insertions. There is no definitive explanation for why RdRp may dissociate from the template at sequences with high PE, but increased structural plasticity in the region may lead to the formation of an ensemble of secondary structures, including ones that disrupt RdRp processivity (40).

All therapeutic siRNA approaches for controlling pathogens suffer from rapid pathogen evolution that reduces siRNA complementarity (41–44). A major advantage of VIGS technology over traditional genetic modification approaches is its flexibility (45), with updated versions ready for deployment within weeks. The evolution of resistant pathogen strains can also be delayed by including multiple siRNA targeting sequences in the VIGS vector and/or designing inserts that target conserved regions of metabolically critical components, which the pathogen cannot easily modify (46). However, the flexibility of VIGS requires the design of insertions that are likely to be stable. Therefore, establishing a strategy for designing stable hairpin inserts for viral vectors marks a significant breakthrough in biotechnology. Our new approach for improving the CY1 VIGS vectors is likely applicable to other VIGS vectors. This would enable detailed gene expression studies and offer innovative solutions for controlling pests and pathogens. Expanding the use of VIGS for tree and vine protection will hopefully encourage researchers to adapt many of the 50 known VIGS vectors to longer-lived plant hosts that were previously considered unfeasible (1, 9, 47, 48). This advancement should have a wide-reaching impact, driving economic growth and increasing global agricultural output.

## MATERIALS AND METHODS

### Plasmid constructs

pUC-CY1, containing CY1 cDNA cloned into pUC19 downstream of a T7 RNA polymerase promoter, and pCB301-CY1, the CY1 cDNA-containing binary vector for *A. tumefaciens* infiltration, were previously described (20). *N. benthamiana* PDS gene (GenBank accession number LC543535.1) fragments PDS60 and PDS500 (positions 441–500 and 441–940, respectively) were amplified from cDNA synthesized from total RNA extracted from *N. benthamiana* leaves. *Nicotiana tabacum* magnesium-chelatase subunit I gene (GenBank accession number NM_001324838.1) fragments ChlI81 and ChlI215 (positions 527–607 and 527–747, respectively) were similarly amplified. Nano-Luciferase sequence (GenBank accession number OKO48808.1) was amplified from a previously constructed plasmid. pUC-CY1 and pCB301-CY1 were linearized using insertion site-generating primer pairs and subsequently assembled with the desired inserts using ligation-independent cloning (49). Natural hairpins H1, H2, H3, and H4 were amplified from CY1 cDNA. Ftsz160, Ftsz159, and Ftszrc160 sequences were synthesized by Synbio Inc. Inserts were assembled into linearized pCB301-CY1 using the NEBuilder HiFi DNA Assembly kit following manufacturer’s instructions (NEB). All constructs were verified by Sanger sequencing.

### *In vitro* translation

pUC-CY1-derived plasmids were linearized with HindIII and used as templates for *in vitro* transcription with T7 RNA polymerase, yielding RNA transcripts with the exact 5' end and a single non-templated nucleotide at the 3ʹ end. The synthesized RNAs were purified by lithium chloride precipitation and normalized using a DeNovix DS-II FX spectrophotometer (50). RNA templates (0.5 pmol) were added to a 10 µL WGE reaction mix (Promega) containing ^35^S-methionine, 5 µL WGE, 0.8 µL 1 mM amino acid mixture (without methionine), and 100 mM potassium acetate. The translation mixture was incubated at 25°C for 45 min. Following incubation, 10 µL of 2× SDS loading buffer (10% glycerol, 2% SDS, 90 mM Tris-HCl pH 6.8, 0.1% bromophenol blue, and 0.1% 2-mercaptoethanol) was added to the reaction mixture, and the samples were subjected to 10% SDS-PAGE. The gel was dried, exposed to a phosphorimager screen, and scanned the next day using a Typhoon image analyzer (Amersham). Bands were quantified using GelQuantNet software.

### Plant growth

*N. benthamiana* plants were grown at 25°C with a 12-hour light period and 70% humidity. Citrus trees were grown at 30°C with a 16-hour light period and 70% humidity.

### Vacuum infiltration of *A. tumefaciens* and RNA extractions

*A. tumefaciens* strain GV3101 was transformed with PCB301-CY1 constructs and cultured in LB medium containing appropriate antibiotics at 28°C with shaking overnight. The culture was centrifuged at 3,000 rpm for 15 min, and pelleted bacteria resuspended in infiltration buffer [10 mM 2-(*N*-morpholino)ethanesulfonic acid [MES], 10 mM MgCl_2_, and 100 µM acetosyringone]. The OD_600_ was adjusted to 1.0 and mixed in a 1:1 ratio with *A. tumefaciens* containing pothos latent virus p14 or tomato bushy stunt virus p19 gene silencing suppressor-expressing plasmid at OD_600_ of 0.4 and incubated at room temperature for 2.5 hours prior to vacuum infiltration. *N. benthamiana* plants with six to eight fully expanded leaves were submerged upside down in the cell suspension, and a vacuum of −25 PSI was applied for 30 s and released. Vacuum infiltration for citrus trees was performed similarly, but with 2 min vacuum exposure, which was repeated until leaves were fully infiltrated. Total RNA from agroinfiltrated plants was extracted using TRIzol reagent (Invitrogen) according to the manufacturer’s instructions.

### RT-PCR screening for insert retention

Total RNA was treated with RQ-1 DNase (Promega) prior to reverse transcription. First-strand cDNA was synthesized using random hexamers and M-MuLV reverse transcriptase (NEB). Primers 5ʹ-CTGTCATGGGCTCGCTGAGT-3ʹ and 5ʹ-CCACAGTGCTATCGCTCCAA-3ʹ, which are located external to all insert sites, were used to amplify positions 2052–2489. PCR products were subjected to electrophoresis through 1% agarose gels and further analyzed on 2% gels if small deletions appeared to be present. Some of the PCR products were subjected to batch sequencing following gel purification.

### MFE or ∆G, PE, and APE values

The RNAfold WebServer (http://rna.tbi.univie.ac.at/cgi-bin/RNAWebSuite/RNAfold.cgi; 2020–2024) was used to obtain structure and thermodynamic information for inserts. Bracket and dot notations, ∆G values, and EPS files were obtained for the MFE structures. For the EPS file, the option “MFE structure drawing encoding positional entropy” was selected (51). Bracket and dot notations were used to draw RNA structures using the RNAcanvas web app (52; https://rnacanvas.app; 2023–2024). PE values for individual nucleotides were extracted from the EPS files and applied to RNAcanvas-drawn structures using the constructed program PJNTrophy for clear to red residue color coding (see data availability). APE values for hairpins and regions of CY1 secondary structure were calculated based on PE values of the individual residues. In this study, the maximum index value for PE was set to 2.6, as this was the highest PE for any residue among all the structures.

### Random hairpin generation and analysis

Using automated string manipulation in Python (version 3.12.3), 38,577 hairpins were randomly generated using various stem lengths (10, 15, 30, 45, 60, 75, or 90 bps), an apical loop of five randomly chosen nucleotides, and varying degrees of complementarity (100%, 90%, 80%, 70%, 60%, 50%, 40%, 30%, 20%, 10%, or 0%). For 100% complementary hairpins, each nucleotide on the 5ʹ end of the stem was paired with its Watson-Crick partner on the 3ʹ end of the stem. For less complementary hairpins, bases on the 3ʹ end of the stem were either perfectly complementary to the corresponding 5ʹ base or were randomly selected regardless of the 5ʹ end such that the overall complementarity could only be confirmed for a given percentage of the bases. For the 0% complementarity hairpins, the 5ʹ and 3ʹ ends were randomly generated independently of each other. Each hairpin was then folded *in silico* using the ViennaRNA package (version 2.6.4), and the thermodynamic properties were compared to the hairpins found in CY1.

### Analysis of hairpins found in other +RNA viruses, DNA viruses, plant mRNA, and ribosomal RNA

Secondary structures for TBSV (23), HCV (21), and human 16S (https://rnacentral.org/rna/URS000035C796/9606) and 28S (https://rnacentral.org/rna/URS0000ABD8B3/9606) ribosomal RNA were searched for hairpins, and the corresponding sequences were folded *in silico* using the ViennaRNA package (version 2.6.4). Plant mRNA from both *Arabidopsis thaliana* and *Nicotiana tabacum* (NM_001035540.3, NM_001085318.5, NM_001125865.3, NM_001203084, NM_001337428.1, NM_001341019.1, NM_001342214.1, NM_001342703.1, NM_001343254.1, NM_001345462.1, NM_111130.3, NM_115393.3, NM_118738.6, NM_119712.6, NM_119996.2, NM_120281.3, NM_120667.5, NM_121392.3, NM_124089.5, NM_125860.5, NM_126153.4, V01481, X54430.1, X56263.1, X60058.1, X77950.1, XM_016577897.1, XM_016621179.1, XM_016652471.1, and Z48977.1) and the coding regions from DNA viruses (human papillomavirus 11 JN644142.1 E1a, E1b, E2, E5, E6, E7a, E7b, L1a, L1b, and L2; BK polyomavirus LC029411.1 VP1, VP2, small T-antigen, large T-antigen; chickpea chlorotic dwarf virus HG934858.1: MP, CP, Rep, Rep-A; tomato yellow leaf curl virus HG969271.1 CP, REn, TrAP, V2, Rep, and C4; wheat dwarf virus MN594280.1 MP, CP, and Rep-A; chilli leaf curl virus MT800761.1 U2, CP, AL2, AL3, and Rep) were first folded as complete structures, which were then searched for simple hairpins and analyzed using the same method as the +RNA virus hairpins.

## Data Availability

The program PJNTrophy and all data found in Fig. 11 are available at 10.6084/m9.figshare.27214308.

## SUPPLEMENTAL MATERIAL

Supplemental figures jvi.01919-24-s0001.pdf Figures S1 to S3.

ASM does not own the copyrights to Supplemental Material that may be linked to, or accessed through, an article. The authors have granted ASM a non-exclusive, world-wide license to publish the Supplemental Material files. Please contact the corresponding author directly for reuse.
