# Supplementary material for: Hairpin inserts in viral genomes are stable when they conform to the thermodynamic properties of viral RNA substructures
Source: J Virol. 2025 Mar 21;99(4):e01919-24. doi: 10.1128/jvi.01919-24 (PMC11998532; doi:10.1128/jvi.01919-24)
Supplement: Supplemental figures — Figures S1 to S3. [file jvi.01919-24-s0001.pdf]

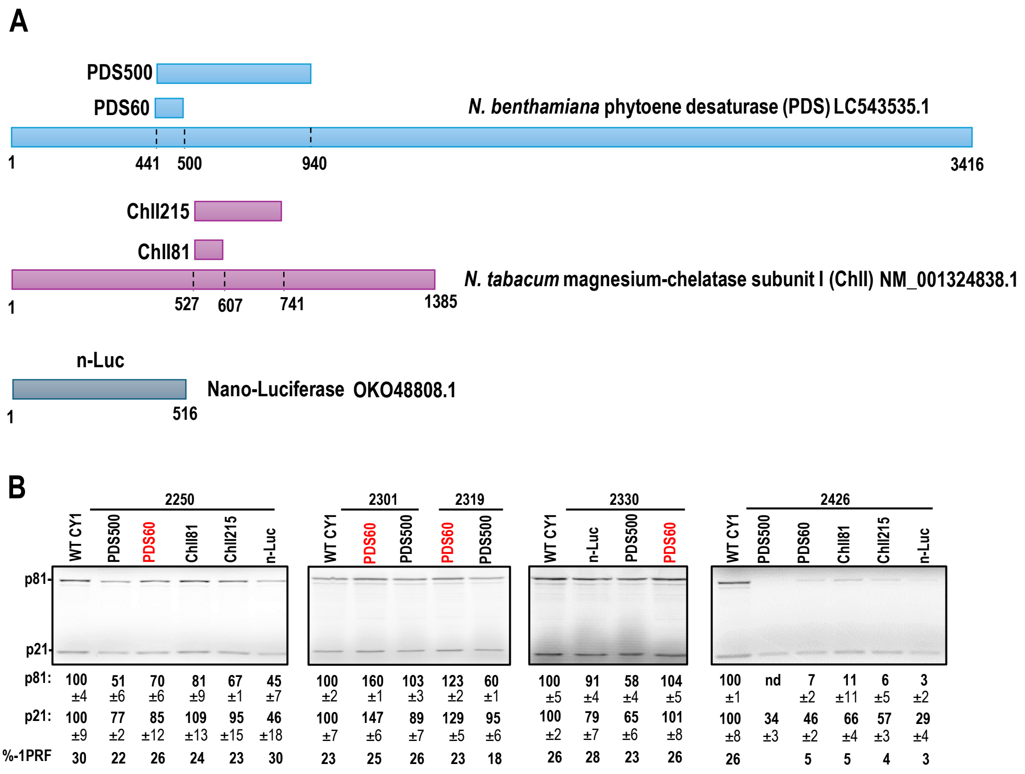

SFig 1 Screening insertion sites in CY1. **(A)** Schematic representation of inserts PDS500, PDS60, ChlI215, ChlI81, and n-Luc that used to screen possible insert sites (numbers reflect insert length). **(B)** In-vitro translation of p21 and p81 using CY1 templates with inserts at the indicated sites. Values of p21 and p81 were normalized as a percentage of WT CY1. Standard deviations are shown for 3 independent experiments. (%)-1PRF was calculated based on the absolute levels of p21 and p81 synthesized for each construct. Red color denotes that only these constructs were infectious in *N. benthamiana*, producing typical early CY1 symptoms of a cupped-shaped leaf and stunting between 14- and 21-days post-infiltration. **(C)** Detection of PDS60$_{2301}$ from symptomatic plants at 3-wpi (lanes 1-4). CY1 was not detected in non-symptomatic plants (lanes 5-7), but agrobacteria-infiltrated local tissue revealed a very faint RT-PCR band (7-L). N: negative control.

GFP59m$_{2304}$*

uuuccguaUGAAGCGGCACGACUUCUUCAAGAGCGAUAACUCGCCUUGACAGAAGUCCAACGCUUCAucuag
uuuccguaUGAAGCGGCACGACUUCUUCAAGAGCGAUAACUCGCCUUGACAGAAGUCCAACGCUUCAucuag

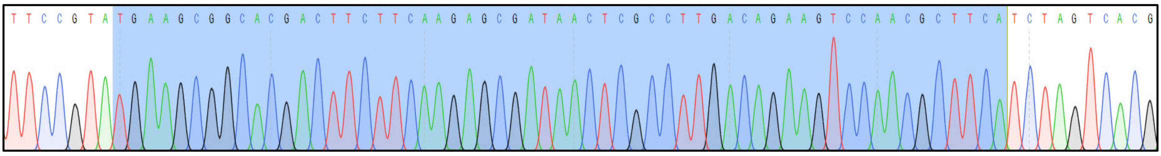

GFP61m$_{2219\Delta H4}$*

uucguUGAAGCGGCACGACUUCUUCAAGAGCGAUAACUGGUGUCUUGACAGAAGUCCAACGCUUCAguuaa
uucguUGAAGCGGCACGACUUCUUCAAGAGCGAUAACUGGUGUCUUGACAGAAGUCCAACGCUUCAguuaa

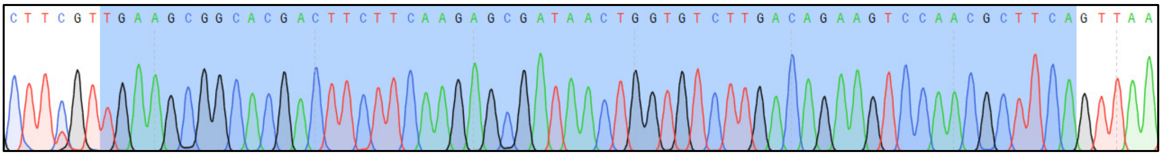

SFig 2 Sequence alignments and batch sequencing chromatograms for GFP59m$_{2304}$∗ (retained) and GFP61m$_{2219\Delta H4}$∗ (retained).

Ftsz159$_{2219\Delta H4}$**

uucguuCUAAGCAGAUUAUUCAACUAGGAUCUGGAAUAACCGAAGGUUUGGGAGCAGGAUCUCAUCCAGAAGUUGGUCGUGCUGCU
ACCAGCGCGACCAACGUCUCGAUGCGAUCAUGCUCACAAACCUUCAAUUAUUCCAGAUCCUAGCCGAAUAAUAUGCUUAGaguuaa

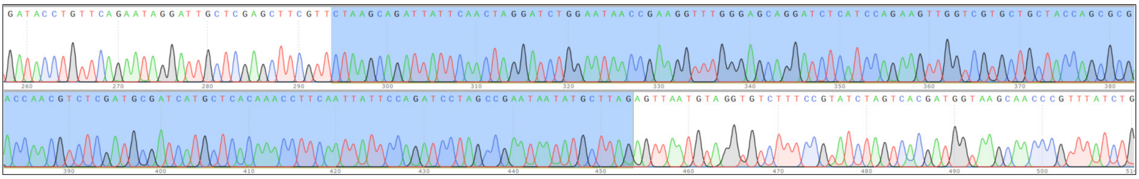

Ftszrc160$_{2219\Delta H4}$**

uucguuCUAGGCAUUAUUAUUUGACUAAGAUCUGUUAUAGUUGAAGGCAGGCCGUAUGAUUUCAUUUACGGGUUGAUUGUGCUUG
UAGCAGCACGACCAACUUCUGGAUGAGAUCCUGCUCCCAAACCUUCGGUUAUUCCAGAUCCUAGUUGAAUAAUCUGCUUAaguuaa

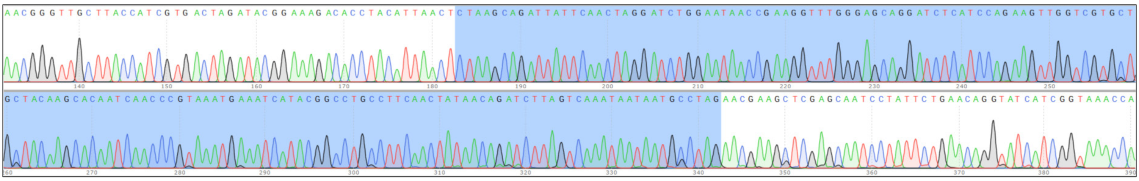

Ftsz160$_{2219\Delta H4}$**

uucguuCUAAGCAGAUUAUUCAACUAGGAUCUGGAAUAACCGAAGGUUUGGGAGCAGGAUCUCAUCCAGAAGUUGGUCGUGCUGCU
uucguuCUAAGCAGAU--

ACCAGCGCGACCAACGUCUCGAUGCGAUCAUGCUCACAAACCUUCAAUUAUUCCAGAUCCUAGCCGAAUAAUAUGCUUAGaguuaa
-- CCUAGCCGAAUAAUAUGCUUAGaguuaa

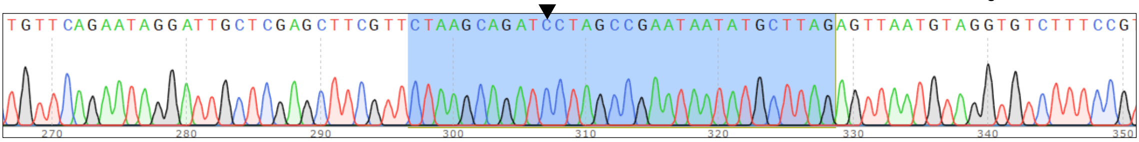

SFig 3 Sequence alignments and batch sequencing chromatograms for Ftsz159$_{2219}$DH4∗ (retained), Ftszrc160$_{2219}$DH4∗ (retained), and Ftsz160$_{2219}$DH4∗ (not retained).
